# Purine nucleoside phosphorylase dominates Influenza A virus replication and host hyperinflammation through purine salvage

**DOI:** 10.1038/s41392-025-02272-1

**Published:** 2025-06-15

**Authors:** Yang Yue, Qingyu Li, Changguo Chen, Juntao Yang, Weian Song, Changdong Zhou, Yuke Cui, Zhenqiao Wei, Qi He, Chenhui Wang, Hongjun Lin, Jiangbo Li, Jian Li, Ji Xi, Xiang Song, Wen Yang, Ze Zhang, Wenjie Shu, Liang Guo, Shengqi Wang

**Affiliations:** 1 Bioinformatics Center of AMMS, Beijing, China; 2 The Sixth Medical Center of Chinese, PLA General Hospital, Beijing, China; 3 State Key Laboratory of Common Mechanism Research for Major Diseases, Institute of Basic Medical Sciences, Chinese Academy of Medical Science and Peking Union Medical College, Beijing, China

**Keywords:** Infectious diseases, Infection

## Abstract

Influenza A virus (IAV) poses a significant threat to human health. The outcome of IAV results from the viral-host interaction, with the underlying molecular mechanisms largely unknown. By integrating the plasma proteomics data of the IAV-infected patients into the viral-inflammation protein-protein interaction (VI-PPI) network created in this study, purine nucleoside phosphorylase (PNP), the critical enzyme in purine salvage, was identified as a potential hub gene that connected the different stages of IAV infection. Extended survival rates and reduced pulmonary inflammatory lesions were observed in alveolar epithelial cell (AEC)-specific PNP conditional knockout mice upon H1N1 infection. Mechanistically, PB1-F2 of IAV was revealed as a novel viral transcriptional factor to bind to the TATA box of PNP promoter, leading to enhanced purine salvage in H1N1-challenged AECs. The activation of PNP-mediated purine salvage was verified in IAV-infected patients and A549 cells. PNP knockdown elicited a purine metabolic shift from augmented salvage pathway to de novo synthesis, constraining both viral infection and pro-inflammatory signaling through APRT-AICAR-AMPK activation. Moreover, durdihydroartemisinin (DHA), predicted by VI-PPI as a novel PNP inhibitor, exerted beneficial effects on the survival and weight gain of H1N1-challenged mice via its direct binding to PNP. To reveal for the first time, we found that PNP, activated by IAV, plays a hub role within H1N1-host interaction, simultaneously modulating viral replication and hyperinflammation through purine salvage. Our study sheds new light on a “two-for-one” strategy by targeting purine salvage in combating IAV-related pathology, suggesting PNP as a potential novel anti-influenza host target.

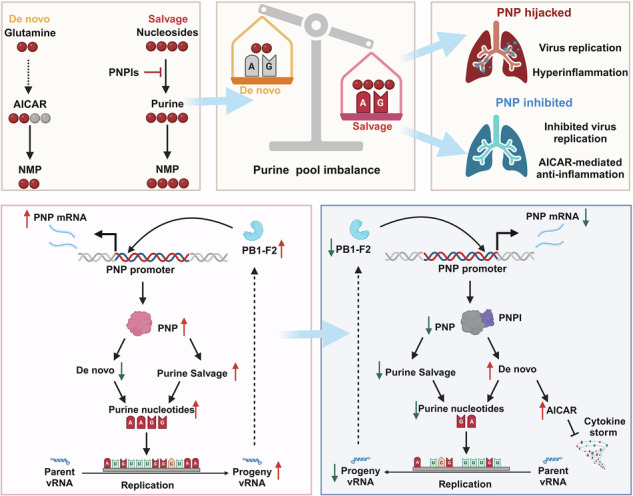

## Introduction

Seasonal influenza A virus (IAV) infections cause up to five million cases of severe illness, leading to a total of 650,000 deaths worldwide annually.^1^ In late 2023 to 2024, highly pathogenic avian influenza (HPAI) A (H5N1) clade 2.3.4.4b viruses were identified in dairy cows and caused dairy workers infected, which is threatening the public health.^2,3^ The outcome of the infection is believed to result from the interplay of viral virulence and host inflammation. In addition, certain IAV strains have been reported to be particularly lethal to younger, ostensibly healthy adults, which is attributed to the strong inflammatory response of the host.^4^ Current therapeutic strategies and vaccines, typically designed to target the virus itself, have limited capacity to inhibit viral replication and may also induce viral resistance.^5^ Noteworthy, dairy cattle HPAI H5N1 has shown resistance to NA (neuraminidase) inhibitors including Oseltamivir phosphate, zanamivir and peramivir.^2^ Moreover, no effective therapies have been developed for IAV-induced hyperinflammation and the related acute respiratory distress syndrome (ARDS).^4^ Consequently, host‐targeting antivirals warrant increased attention, since the host‐directed pathways and molecules hijacked by viruses offer a broad‐spectrum of potential targets for concurrently combating viral infection and mitigating excessive inflammation.^6,7^

To effectively discover genes that serve as intermediaries in the viral-inflammation nexus, there is an urgent need for an advanced screening model to elucidate the dynamic interrelations among genes. Extensively accumulated protein-protein interaction (PPI) data are widely exploited for elucidating intricate connections among molecules.^8–11^ Up to date, the PPI network is typically constructed on the basis of differentially expressed genes (DEGs) obtained from a specific pathological phase, with genes of interest ranked by network centrality.^12,13^ However, during the progression of viral infection, molecules serving as hubs to connect viral infection, host inflammation, and related pathologies might be ignored due to their less neighborhoods and sometimes less pronounced expression changes. And thus, to uncover these obscured yet vital effectors, an intact inflammatory PPI network integrated by sub-PPI modules of different pathological stages of IAV infection should be more efficacious.

Viruses rely on host cell energy machinery and usurp host metabolic resources to fuel viral replication and transmission.^14,15^ Among multiple virus-reprogramed cellular metabolic pathways, nucleotide metabolism has garnered great attention due to its critical role in virion production and other steps of viral life cycle.^16^ Purine and pyrimidine are synthesized through either de novo or salvage pathways, and the inhibitors for pyrimidine and purine pathways targeting rate-limiting enzymes have been proposed as potential antivirals.^17,18^ Of note, previous studies have mainly focused on de novo synthesis rather than salvage pathways in viral infection, since methods for measuring nucleotide salvage were not well established.^19^ Actually, perturbation of purine salvage was reported in the intestinal cells of C. elegans to increase resistance to intracellular and extracellular pathogen infection.^20^ More recently, both purine and pyrimidine salvage pathways have been identified to modulate the infection of picornaviruses.^21^ During IAV infection, the cellular metabolism of purines and pyrimidines are dysregulated,^22^ yet neither the imbalance between the host de novo and salvage pathways, nor their roles in the progression of the disease, has been investigated.

Among the various critical enzymes of nucleotide metabolism, purine nucleoside phosphorylase (PNP), an evolutionarily conserved and key enzyme of purine salvage,^23^ has been reported to affect the viability of various of pathogenic organisms, including *Mycobacterium tuberculosis* and *Plasmodium falciparum*.^24^ Furthermore, PNP is related to normal functioning of host immunity. PNP deficiency in human induced severe T-cell dysfunction,^20^ and PNP inhibitors have been employed as selective immunosuppressive agents for T-cell malignancies and T-cell mediated autoimmune diseases.^25^ However, little is known about the potential roles of PNP-mediated salvage pathway in the viral-host interplay during IAV infection.

In this study, to efficiently explore host targets serving as intermediaries between influenza infection and inflammation, we created a viral-inflammation (VI)-PPI network based on human proteins involved in viral infections, lung inflammation, and drug targets from multiple published datasets. By integrating the plasma proteomic profiling data of H1N1-infected patients within the VI-PPI network, PNP was identified. PB1-F2 protein of H1N1 was revealed, for the first time, as a viral transcription factor to bind the TATA box of PNP promoter predicted by Alphafold 3, leading to enhanced purine salvage pathway in H1N1-infected AECs and patients. PNP knockdown reverted purine synthesis to de novo pathway to suppress viral replication and host inflammation through AMP-activated protein kinase (AMPK) signaling that was activated by 5-aminoimidazole-4-carboxamide ribonucleotide (AICAR, an intermediate metabolite of de novo purine synthesis). Additionally, extended survival rates and reduced pulmonary inflammatory lesions were observed in the infected AEC-specific PNP conditional knockout mice. Moreover, dihydroartemisinin, predicted by VI-PPI network as a novel PNP inhibitor, was validated to directly bind PNP to efficiently combat H1N1 infection. In conclusion, it is for the first time that host PNP is revealed to be transcriptionally activated by the viral PB1-F2 protein and play hub roles in modulating viral replication and hyperinflammation through purine salvage.

## Results

### PNP was identified to play hub roles in viral-host interaction upon H1N1 infection

**Fig. 1:**
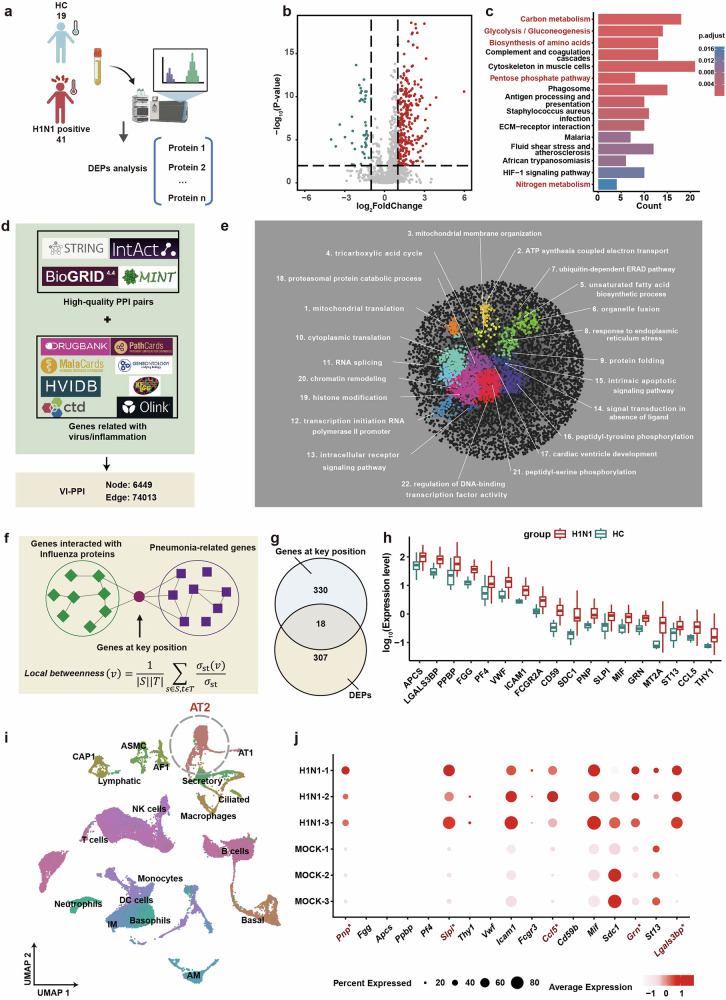
PNP was identified as a potential hub gene in IAV infection via VI-PPI network. **a** Overview of the workflow for the proteomic analysis of the plasma derived from H1N1-infected patients and healthy controls. The peripheral plasma of 19 healthy controls and 41 H1N1-infected patients were collected, followed by proteomics analysis. **b** The volcano map of the plasma proteomic data. The differentially expressed proteins (DEPs) were labeled as blue (downregulation) and red (upregulation). *P* < 0.05, fold change > 1.5 or <0.6667. **c** The KEGG pathway enrichment analysis of DEGs between the healthy and H1N1-infected individuals. The top 15 KEGG pathways enriched by DEGs (H1N1-infected vs. Healthy controls) were displayed. **d** Data collection for VI-PPI. VI-PPI network was constructed on the basis of protein-protein interaction, containing molecules related to viral infection, host inflammation, and drug targets, with 6449 nodes (genes) and 74103 edges (PPI pairs). **e** The landscape of VI-PPI. A total of 22 functional domains were enriched and marked with different colors by SAFE tools in Cytoscape. Each cluster was annotated with the most relevant gene ontology (GO) term. **f** The algorithm for the identification of the key hub genes during influenza progression. Hub genes by VI-PPI network, defined as genes that were significantly located at the shortest paths from H1N1-related genes to pneumonia-related genes, were extracted by calculating the *local betweenness centrality* (the upper panel). **g** The Venn plot of shared hub genes among infection of different strains of influenza virus by VI-PPI. 348 hub genes with potential broad-spectrum among the 4 strains of influenza virus (H1N1, H7N7, H5N1, H3N2, Supplementary Fig. 1e) by VI-PPI prediction were used to overlap with a total of 325 differentially expressed proteins (DEPs) derived from the plasma proteome of clinical cohort (H1N1 infected vs. healthy control). 18 overlapped genes were identified by Venn plot. **h** The overview of the expression of the overlapping genes by the plasma proteomic data of the clinical cohort. The expression of the 18 overlapping DEPs based on the proteomic data of H1N1-infected vs. healthy control was shown by box plot. **i**, **j** The ScRNA-seq analysis of lungs derived from the control and H1N1 mice models. The scRNA-seq data of H1N1-infected mice were obtained from previously published data (https://ngdc.cncb.ac.cngsabrowse/CRA013573). t-SNE plot revealed 19 clusters, and the expression of the 18 overlapping DEPs in alveolar type 2 (AT2) cells was analyzed. Differentially expressed genes (DEGs) with significance were labeled as red. All data are presented as mean ± SD; unless otherwise indicated, *N* = 3 biologically independent experiments; Statistical analysis was performed by two-tailed Student’s *t*-test (**h**) and Wilcoxon rank-sum test (**j**). ^*^P < 0.01 versus Mock (**j**)

In order to discover novel regulators within the viral-host interaction, the peripheral plasma of 41 IAV-infected patients and 19 healthy volunteers were collected and subjected to proteomics analysis (Fig. 1a). Compared to the healthy controls, 325 differentially expressed proteins (DEPs) were identified in IAV-infected patients, which were enriched in multiple metabolic pathways based on Kyoto Encyclopedia of Genes and Genomes (KEGG) functional enrichment analysis (Fig. 1a, c).

To efficiently explore nexus molecules within the viral-host interaction during IAV infection from a large quantities of candidate molecules, we firstly created a screening model called Viral-Inflammation Protein-protein Interaction network (VI-PPI), through manually collecting genes related to viral infection and host inflammation, as well as high-quality physical PPI pairs, from multiple published datasets (Fig. 1d, e, Supplementary Fig. 1a, b, and Supplementary Table 1). As shown in Fig. 1e, all the nodes in VI-PPI were enriched in pathways associated with fundamental homeostasis maintenance function based on SAFE analysis (Supplementary Table 2), yielding a scale-free network,^26^ with higher density compared to other published PPI networks (Supplementary Fig. 1c, d). Secondly, we proposed a novel strategy for screening potential host targets with hub roles. The hub genes were defined as genes that exhibited significance calculated by *local betweenness centrality* (see Methods) and located at the shortest paths between host factors interacting with IAV proteins and molecules related to pneumonia. Then, to gain insights into broad-spectrum host targets, hub genes that might function during the infection of four subtypes of IAV, including H5N1 (A/goose/Guangdong/1/1996), H3N2 (A/NewYork/392/2004), and H7N7 (A/mallard duck/Korea/VI142063/2014), were screened via VI-PPI network, and 348 shared genes were identified (Supplementary Fig. 1e).

Furthermore, by comparing the DEPs lists from IAV-infected patients with the 348 shared genes (Fig. 1f), 18 overlapping genes were identified (Fig. 1g, h). Since alveolar type II (AT2) cells were the primary targets for IAV, we further focused on the change of the 18 genes in AT2 cells by the previously published scRNA-seq data of the lungs derived from the control and H1N1-infected mice (Fig. 1i).^27^ Galectin 3 binding protein (LGALS3BP), granulin precursor (GRN), C-C motif chemokine ligand 5 (CCL5), secretory leukocyte peptidase inhibitor (SLPI), and purine nucleoside phosphorylase (PNP) were revealed with the most significant variations (Fig. 1j). LGALS3BP, GRN, CCL5, and SLPI have been suggested as anti-IAV targets,^28–31^ yet no data has been reported regarding the potential role of PNP in IAV infection. Moreover, since the investigation of purine salvage, an energy-efficient manner for nucleotides production, has not been adequately addressed, PNP, as the critical enzyme in purine salvage, was selected for further study.

### PNP contributed to H1N1 replication in AECs both in vitro and in vivo

**Fig. 2:**
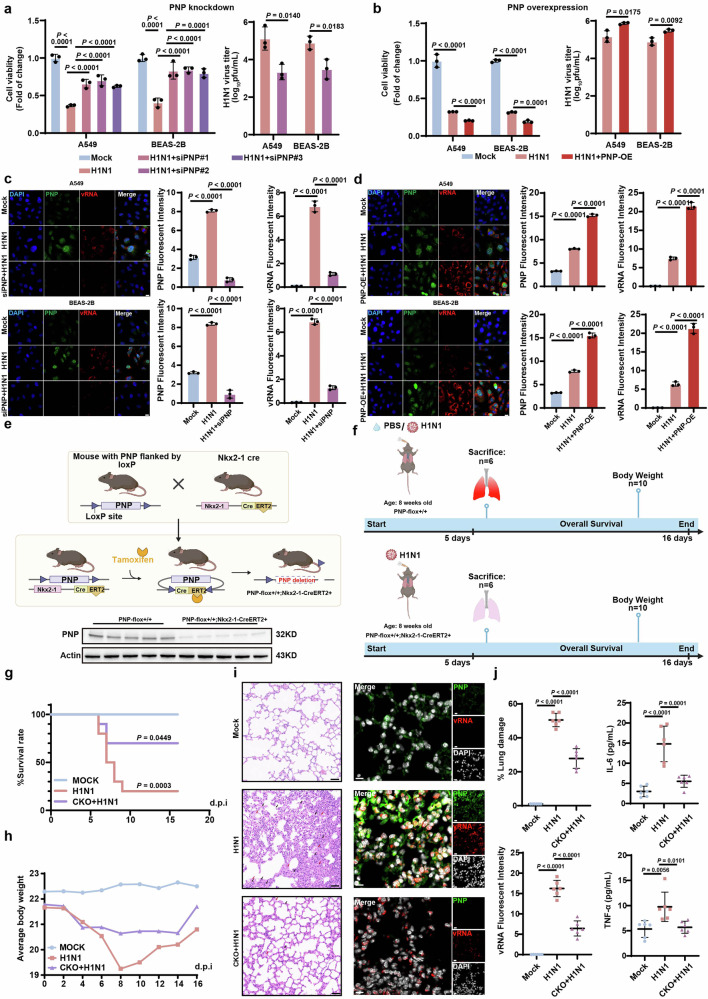
PNP knock down/knock out in alveolar epithelial cells (AEC) exhibit anti-influenza capacity in vitro/in vivo. The effect of PNP inhibition (**a**) or PNP overexpression (**b**) on cell viability and virus titer upon 24 h post H1N1 infection. A549 or BEAS-2B cells were transfected with NC/PNP siRNA at 30 pmol (**a**) or 2 µg control vector/PNP-overexpression vector (**b**) before challenged with H1N1 (MOI = 5). Cell viability and viral titer of H1N1-infected A549 cells and BEAS-2B cells with or without PNP knockdown/overexpression were evaluated at 24 h post-infection. FISH and IF analysis of vRNA and PNP expression in alveolar epithelial cells with or without PNP knockdown/overexpression upon H1N1. Both A549 and BEAS-2B cells were transfected by NC/PNP siRNA (**c**) or control vector/PNP-overexpression vector (**d**), followed by FISH of H1N1 viral genomic RNA (vRNA) and IF assay of PNP, respectively. DAPI was used to stain nuclei of the cells (Scale bar: 20 μm). **e** Schematic representation of the generation of AEC-specific PNP conditional knockout mice models. PNP expression in the lung tissue of PNP^flox/flox^ and Nkx2-1-CreERT2, PNP^flox/flox^ mice was analyzed by Western blot. **f** Diagram of the experimental procedures. Briefly, 8-week-old PNP^flox/flox^ mice and PNP conditional knockout mice were intranasally challenged with 2LD_50_ (**h**) or 4LD_50_ (**g**) H1N1. The survival rate and body weight change upon IAV infection. The survival rate (**g**) and body weight change (**h**) of the different groups (*n* = 10) were observed daily for a course of 16 days. The inflammation lesion and viral infection within the lungs. After 6 days of infection, 6 mice from each group were sacrificed, with the lungs dissociated and subjected to H&E staining (*N* = 6, Scale bar: 50μm, (**i**), FISH of vRNA and IF assay of PNP. DAPI was used to indicate nucleus (*N* = 6, Scale bar: 10 μm, **i**). The area of lung damage and intensity of vRNA were shown. The concentration of TNF-ɑ and IL-6 levels in mice plasma (*N* = 6) was measured by ELISA on day 6 post infection (**j**). All data are presented as mean ± SD; unless otherwise indicated, *N* = 3 biologically independent experiments; Statistical analysis was performed by one-way ANOVA (**c**, **d**, **j**), two-tailed Student’s t test (**a**, **b**) and Log-rank test (**g**)

Furthermore, the expression of PNP in the lung tissues of mice infected with H1N1, H3N2, and H5N1 viruses, respectively, were analyzed, showing a significant increase at both the mRNA and protein levels (Supplementary Fig. 1f). We then evaluated the effect of PNP on H1N1-infected AECs by interfering PNP expression through either siRNA transfection or PNP-overexpression vector (Supplementary Fig. 1g, h). PNP knockdown significantly decreased the viral titer and enhanced cell viability in response to H1N1, compared to the mock-infected cells, whereas overexpression of PNP increased the viral titer and reduced cell viability (Fig. 2a, b). We further employed fluorescence in situ hybridization assay (FISH) assays to verify the association of PNP with viral replication. Inhibition of PNP significantly diminished viral genomic RNA (vRNA) expression in both challenged A549 and BEAS-2B cells, whereas overexpression of PNP increased vRNA expression (Fig. 2c, d).

Next, we validated the in vivo role of PNP during IAV infection. Tamoxifen (TAM)-inducible AEC-specific homozygous PNP conditional knockout (CKO) mice (Nkx2-1-CreERT2, PNP^flox/flox^) were established by mating Nkx2-1-CreERT2 mice with PNP^flox/flox^ mice (Fig. 2e). TAM (75 mg/Kg) was administered to 6-week-old PNP-CKO and PNP^flox/flox^ mice through intraperitoneal injection once a day for 6 consecutive days. One week later, PNP deficiency in lung was determined by Western blot (Fig. 2e). PNP-CKO mice intranasally infected with H1N1 (Fig. 2f) displayed a significantly extended survival rate and an accelerated body weight restoration when compared to PNP^flox/flox^ mice (Fig. 2g, h). Pulmonary pathological injury was also alleviated in PNP-CKO mice at 6 days post-infection (dpi), evidenced by reduced hemorrhage, necrocytosis of epithelial cells, infiltration of inflammatory cells within the lung tissues, and suppressed proinflammatory cytokines production (IL-6, TNF-ɑ) in the peripheral blood (Fig. 2i, j). Moreover, vRNA was significantly diminished in the lungs of PNP-CKO mice by FISH analysis (Fig. 2i, j).

Taken all these lines of evidence together, our results indicated that the VI-PPI-predicted PNP played critical roles in H1N1 infection in both in vivo and in vitro models.

### PB1-F2 of IAV acted as a novel vTF to activate host PNP by TATA box binding

**Fig. 3:**
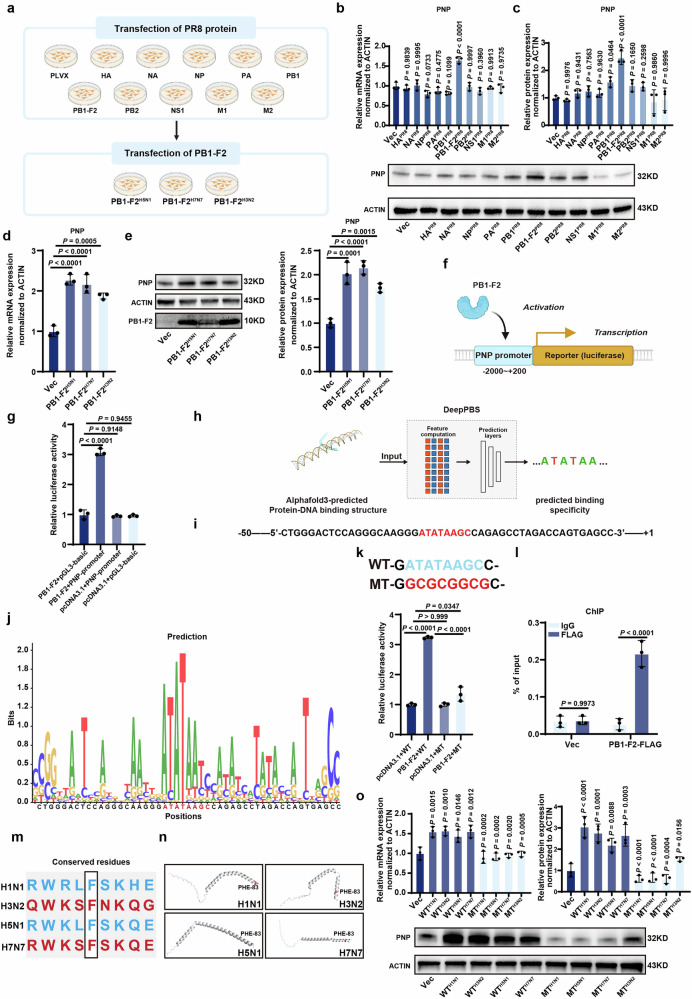
IAV PB1-F2 transcriptionally activated PNP expression by binding to the TATA-box of the promoter. **a** Schematics of the establishment of A549 cells stably transfected by various of H1N1 proteins and the transient transfection of PB1-F2-overexpressing vectors to A549 cells. A549 cells stably overexpressed different H1N1 proteins were used to screen H1N1 protein most relevant to PNP expression. Moreover, PB1-F2-overexpressing vectors of other strains of IAV (H5N1, H7N7, H3N2) were transiently transfected into A549 cells to analyze their effect on PNP expression. The relative expression of PNP in A549 cells stably expressed different H1N1 proteins. The mRNA (**b**) and protein (**c**) level of PNP were quantified by qRT-PCR and Western blot, respectively. The effect of PB1-F2 proteins of other IAV strains ((H5N1, H7N7 and H3N2)) on PNP expression. A549 cells were transiently transfected with vectors overexpressing PB1-F2 of other strains of IAV for 24 h, followed by examining the mRNA (**d**) and protein (**e**) level of PNP via qRT-PCR and Western Blot analysis, respectively. **f**, **g** Effect of PB1-F2 on the luciferase activity of PNP-promoter reporter gene by the dual luciferase assay. The PB1-F2-overexpressing plasmid was co-transfected with pGL3-PNP-promoter reporter gene and Renilla luciferase reporter in 293 T cells for 24 h. Renilla luciferase activities were used as an internal reference. The assay was performed in triplicate. The values are expressed as mean ± SD. **h** Diagram of motif prediction based on Alphafold 3 and DeepPBS. The exact binding site of PB1-F2 on PNP promoter was predicted by combining Alphafold 3 and DeepPBS analysis. **i** Schematics of the −50 ~ + 1 sequence of PNP promoter. The TATA-box was shown in red. **j** Predicted PB1-F2-binding motif on PNP promoter within the −50 ~ + 1 sequence. The PB1-F2-binding consensus sequence is shown in red. **k** Dual luciferase assay of the wild type or TATA-box-mutated PNP luciferase reporter plasmid upon PB1-F2 overexpression. The PNP luciferase reporter plasmid containing the predicted motif (50 bp) with or without the mutated TATA-box were co-transfected with PB1-F2-expressing plasmid and Renilla luciferase reporter into 293T cells for 24 h. Renilla luciferase activities were used as an internal reference. The assay was performed in triplicate. The values are expressed as mean ± SD. **l** The binding of PB1-F2 to the TATA-box in PNP promoters was examined by ChIP-qPCR. 293T cells were transfected by PB1-F2-Flag overexpression plasmid and luciferase reporter gene for PNP promoter, followed by anti-Flag antibody pulldown (IgG was used as negative control) and subsequent ChIP-qPCR analysis. ChIP-qPCR was conducted using primers flanking TATA-box in PNP promoters. The occupancy of PB1-F2 on the binding site was calculated as percentage of respective input DNA concentration. **m** Conserved phenylalanine residues among different strains of IAV. Phe-83 was identified to be conserved within PB1-F2 protein across H1N1, H3N2, H5N1, and H7N7 viruses. **n** The structure of PB1-F2 of different strains of viruses was predicted using AlphaFold 3. The phenylalanine residue was highlighted in red. **o** F83Y-mutated PB1-F2 overexpression plasmids of different strains of viruses failed to upregulate PNP expression. A549 cells were transiently transfected with vectors overexpressing either wild-type or mutated PB1-F2 proteins of various strains of IAV for 24 h, followed by examination of the mRNA and protein level of PNP via qRT-PCR and Western Blot analysis, respectively. All data are presented as mean ± SD; unless otherwise indicated, *N* = 3 biologically independent experiments; Statistical analysis was performed by one-way ANOVA (**b**–**e**, **g**, **k**, **o**), two-way ANOVA (**l**)

Subsequently, we tried to unveil the molecular mechanism of H1N1-induced PNP upregulation. The genome of viruses encodes multiple proteins to strictly control viral replication and transmission to other host cells, among which viral transcription factors (vTFs) deserve more attention as they were responsible for regulating host genes.^32^ To reveal the potential association between H1N1 proteins and host PNP, A549 cell lines stably expressing the control pLVX-Puro vector and various of H1N1 protein (HA, NA, NP, PA, PB1, PB1-F2, PB2, NS1, M1, M2) were established (Fig. 3a). The mRNA and protein level of PNP in these cell lines were analyzed, showing that PB1-F2 overexpression led to a significant increase of PNP expression (Fig. 3b, c). Moreover, the PB1-F2-overexpressing vector for H5N1, H3N2, and H7N7 which were also used in the screening of the broad‐spectrum target in the VI-PPI network (Supplementary Fig. 1e) was transfected into A549 cells, respectively, revealing that the increase of PNP by viral PB1-F2 was universal among different subtypes of IAV (Fig. 3d, e).

Furthermore, the potential of PB1-F2 as a novel viral transcription factor for PNP was analyzed by dual luciferase gene reporter assay. The luciferase reporter containing the full-length of PNP promoter (pGL3-PNP-promoter) was constructed and then co-transfected with Renilla luciferase reporter and the control or PB1-F2 overexpressing vector into 293 T cells (Fig. 3f). The pGL3-PNP-promoter was significantly activated by PB1-F2 overexpressing vector (Fig. 3g). To verify the exact binding site of PB1-F2 on PNP promoter, gene fragments of 50 bp in length, taken at 25 bp intervals upstream of the transcription start site (TSS) of PNP, were analyzed by AlphaFold 3^33^ model for predicting the structure of the PB1-F2-DNA complex and DeepPBS, a geometric deep-learning model designed to predict binding specificity from protein-DNA structure,^34^ was used to identify the predicted binding motif between PB1-F2 and PNP promoter (Fig. 3h). Our data indicated that PB1-F2 closely interacted with the 50-bp DNA fragment nearest to the TSS site (−50 ~ + 1) of PNP promoter (Fig. 3i). A TATA-box (located at 20 ~ 30 bp upstream TSS) within the predicted motif was most likely to be the binding core of PB1-F2-DNA complex (Fig. 3j).

To prove this, we constructed luciferase reporter plasmids containing the predicted motif (50 bp) of PNP promoter with or without the mutated TATA-box and found that PB1-F2 overexpression was unable to activate the reporter gene with a mutated TATA box (Fig. 3k). By ChIP-qPCR, the enriched signal of PB1-F2 was also found to be specific within the predicted binding motif of PNP (Fig. 3l). Previous studies have indicated that viruses may possess a universally conserved residue (Phe) within their TATA-box-binding protein-like domain.^35^ We found that Phe-83 of PB1-F2 was conserved in H1N1, H3N2, H5N1, and H7N7, and was likely to be involved in DNA binding Fig. 3m). Furthermore, the structure of PB1-F2 in each viral strain was predicted using AlphaFold 3, revealing that Phe-83 was situated in the alpha helix of PB1-F2 (Fig. 3n). To test the functional significance of this residue, we constructed PB1-F2 overexpression plasmids harboring the F83Y mutation. Our results demonstrated that this mutation significantly diminished the upregulation of PNP, supporting that Phe-83 within PB1-F2 across different viral strains was critical for its interaction with PNP (Fig. 3o).

To our knowledge, our data is the first to reveal that H1N1 protein PB1-F2 serves as a viral transcription factor to transcriptionally activate the expression of host PNP, contributing to the viral-host interaction to promote disease progression.

### PNP knockdown suppresses viral replication via compromised purine salvage

**Fig. 4:**
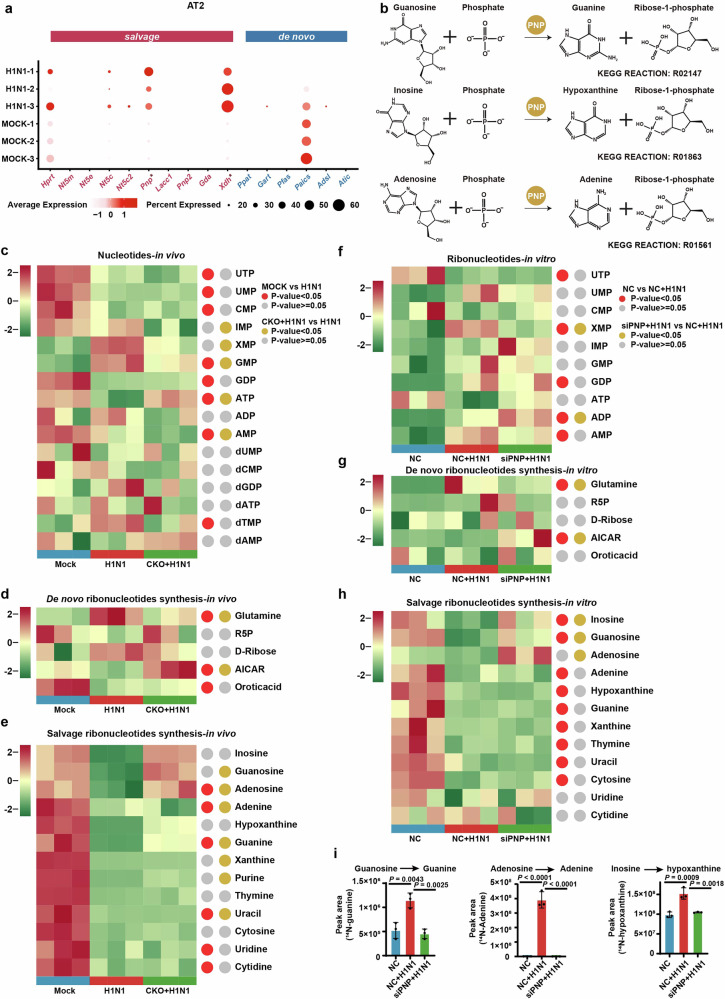
Compromised PNP activity suppresses H1N1-hijacked host purine nucleotide salvage. **a** (ScRNA-seq analysis of lungs derived from control and H1N1 mice models. The expression of enzymes involved in both purine de novo and salvage biosynthesis in alveolar type 2 (AT2) cells. **b** Schematic of PNP-catalized purine salvage pathway. **c**–**e** LC-MS-based targeted metabolomics analysis of AEC-specific PNP conditional knockout mice after challenged for 6 days. Heat map showing the pulmonary metabolites landscape of nucleotides biosynthesis (**c**), dedes synthesis (**d**), and salvage pathway (**e**) based on metabolomic data. The experiments are performed in 3 replicates. Metabolites with *P* < 0.05 were defined as biologically significant. *N* = 3. **f**–**h** LC-MS-based targeted metabolomics analysis of A549 cells with or without PNP knockdown after challenged for 24 h. A549 cells, transfected by 30 pmol NC or PNP siRNA for 12 h, were challenged with H1N1 at an MOI of 5 for another 24 h, followed by metabolomic analysis. Heat map showing the intracellular metabolites landscape of ribonucleotides biosynthesis (**f**), de novo ribonucleotides synthesis (**g**), and salvage pathway (**h**) based on metabolomic data. The experiments are performed in three replicates. Metabolites with *P* < 0.05 were defined as biologically significant. **i** PNP convert purine nucleosides to purines upon H1N1 infection. A549 cells were treated with [U-^15^N] guanosine, [U-^15^N] adenosine and [U-^15^N] inosine for 4 h, respectively. Intracellular ^15^N-Guanine, ^15^N-adenine and ^15^N-hypoxanthine were measured with LC-MS. All data are presented as mean ± SD; unless otherwise indicated, *N* = 3 biologically independent experiments; Statistical analysis was performed by two-tailed Student’s *t*-test (**a**) and one-way ANOVA (**c**-**i**). ^*^P < 0.01 versus Mock (**a**)

As PNP is a critical enzyme of purine salvage, we next investigated the alterations of host purine nucleotide metabolism (PNM) during H1N1 infection. Accordingly, by analyzing the scRNA-seq data of the lungs of IAV-infected mice, expression of multiple purine salvage enzymes, especially PNP, were identified to be upregulated in alveolar type II (AT2) cells during H1N1 infection, while phosphoribosylaminoimidazole succinocarboxamide synthetase (*Paics*), a de novo purine biosynthetic enzyme, was slightly downregulated (Fig. 4a). In line with AT2 cells, AT1 cells had PNP expression dramatically upregulated upon H1N1 (Supplementary Fig. 2a). These results strongly suggested that H1N1 enhanced the activity of host purine salvage in alveolar epithelial cells, in which PNP played a critical role.

We further carried out LC-MS-based targeted metabolomic analysis of PNP-CKO mice and A549 cells, in order to quantify the change of metabolites involved in nucleotide metabolism. We found that IAV infection robustly induced nucleotide metabolism rewiring, and PNP inhibition potently reversed the metabolic disorders (Fig. 4c-h). Particularly, though both purine and pyrimidine ribonucleotide biosynthesis pathways were dramatically affected after H1N1 infection, PNP suppression tended to rescue ATP over-consumption in purine nucleotide metabolism rather than pyrimidine ribonucleotide biosynthesis (Fig. 4c, e). Of note, inosine monophosphate (IMP) was not remarkably changed after H1N1 infection (Fig. 4c, f), suggesting a balance between the consumption and production of IMP in purine synthesis. This balance might be attributed to a host ‘shut-off’ activity of H1N1 to salvage nucleotide supply for viral replication at the price of the reduced synthesis of cellular mRNA, which has been reported in infection of severe acute respiratory syndrome (SARS) coronaviruses.^36,37^

One of the most striking changes in H1N1-infected A549 metabolomics was the reduction of purine salvage nucleosides (guanosine; inosine; adenosine) and the accumulation of de novo purine synthesis substrates (glutamine) (Fig. 4d, g). As salvage pathway can restrain the use of ATP needed for de novo synthesis and ensure efficient usage of recycled nucleobases and nucleosides,^21^ our results indicated that H1N1 infection re-routes host purine metabolism to achieve rapid replication via salvage. Conversely, PNP knockdown by siRNA (siPNP) resulted in an increased level of guanosine, inosine, and adenosine and a reduction of glutamine upon infection (Fig. 4e, h). Moreover, an upregulation of a de novo purine synthesis intermediate 1-(5’-Phosphoribosyl)-5-amino-4-imidazolecarboxamide (AICAR) was observed in infected CKO mices and infected siPNP cells (Fig. 4d, g). Thus, our data indicated that purine salvage pathway in AECs was dramatically activated by H1N1 infection, which could be switched to de novo synthesis via PNP deficiency.

Of note, in eukaryotic cells, adenosine was not considered as the principal substrate of PNP (Fig. 4b).^38^ LACC1 (laccase domain containing (1) has been reported to combine activity analogous to PNP in catalyzing the phosphorolysis of adenosine, guanosine, and inosine.^39^ However, LACC1 was almost non-detectable in AT2 cells (Fig. 4a). As shown in Fig. 4e, h, PNP knockdown restored H1N1-induced adenosine downregulation, and thus we speculated that the massive nucleosides demand of H1N1 replication directly promoted the conversion of adenosine by PNP. We found that, similar to the change of metabolomic analysis, H1N1 markedly increased the accumulation of ^15^N-guanine, ^15^N-adenine, ^15^N-hypoxanthine in A549 cells, which can be reversed by PNP knockdown (Fig. 4i).

Collectively, our results revealed that IAV infection controlled the host purine metabolism through PNP, and PNP deficiency significantly inhibited viral replication by reducing the activity of H1N1-enhanced purine salvage.

### PNP inhibition re-routes metabolic flux from purine salvage to de novo synthesis during IAV infection

**Fig. 5:**
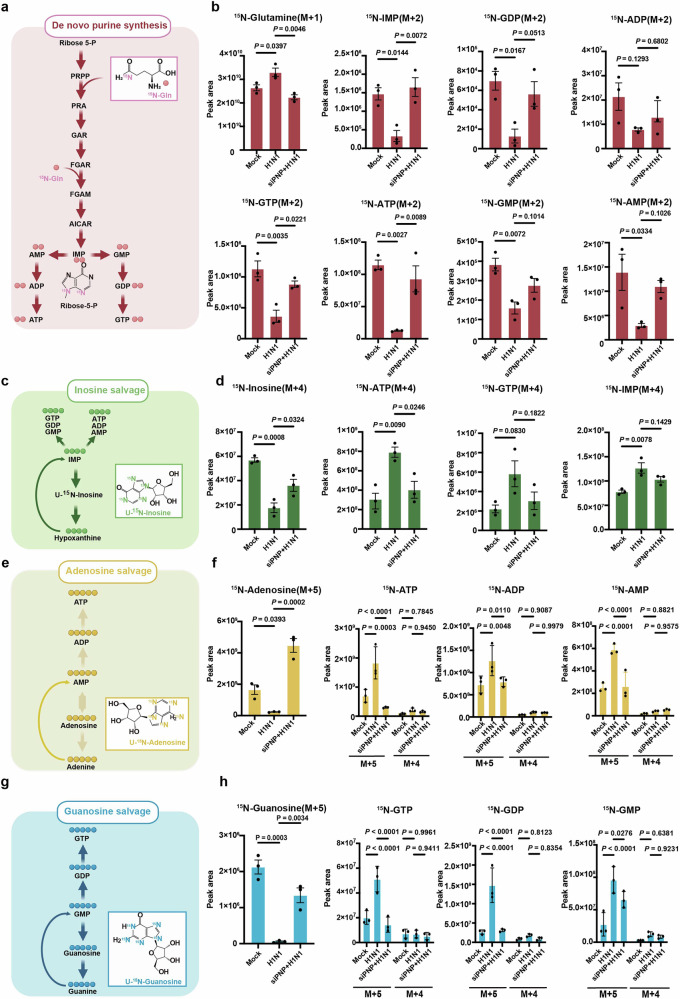
PNP knockdown re-routes metabolic flux from purine salvage to de novo synthesis upon IAV infection. **a**, **c**, **e**, **g** Schematic of metabolic flux assay for purine synthesis. The de novo purine synthesis was tracked by isotype-labeled [Amide-^15^N] glutamine (**a**), inosine salvage by [U-^15^N] inosine (**c**), adenosine salvage by [U-^15^N] adenosine (**e**), and guanosine salvage by [U-^15^N] guanosine (**g**). **b**, **d**, **f**, **h** Metabolic flux analysis of metabolites involved in de novo purine synthesis and purine salvage pathways. The control and H1N1 infected A549 cells were cultured in media containing ^15^N-labeled starting material for 4 h before mass spectrometry analysis of isotype-labeled glutamine (**b**), inosine (**d**), adenosine (**f**), and guanosine (**h**), as well as related purine nucleosides. The experiment was conducted in triplicate. M + (n): the gross (n) of incorporated ^15^N. All data are presented as mean ± SD; unless otherwise indicated, *N* = 3 biologically independent experiments; Statistical analysis was performed by one-way ANOVA (**b**, **d**), **f** (^15^N-Adenosine) and **h** (^15^N-Guanosine); Two-way ANOVA in **f** (^15^N-ATP, ^15^N-ADP and ^15^N-AMP) and **h** (^15^N-GTP, ^15^N-GDP and ^15^N-GMP)

To illuminate the effect of PNP on H1N1 infection-altered host nucleotide metabolism more straightforwardly, metabolic flux analysis was performed to trace the source of the purine ring in the production of ATP and GTP in A549 cells and BEAS-2B cells. H1N1-infected A549 and BEAS-2B cells were incubated with stable isotope-labeled ^15^N-amide-glutamine, U-^15^N inosine, U-^15^N adenosine, and U-^15^N guanosine, all of which contributed to the purine ring synthesis. The peak area of ^15^N-glutamine, the principal substrate of purine de novo synthesis, was much higher in infected cells when compared to the mock, revealing a decreased utilization of ^15^N-glutamine (Fig. 5a, b and Supplementary Fig. 8), while PNP inhibition enhanced the utilization of ^15^N-glutamine in challenged A549 and BEAS-2B cells (Fig. 5b and Supplementary Fig. 8). Accordingly, the peak area of ^15^N-labeled purine intermediates (IMP, AMP, ADP, ATP, GMP, GDP, GTP) during de novo synthesis were decreased in response to H1N1 infection, yet significantly increased by PNP knockdown (Fig. 5b and Supplementary Fig. 8). These results indicated that H1N1 inhibited de novo purine synthesis in alveolar epithelial cells, which could be reversed by PNP suppression.

We next explored the effect of H1N1 infection on purine salvage. In contrast to its restriction on de novo pathway, H1N1 infection increased the consumption of U-^15^N inosine and the generation of ^15^N-labeled purine intermediates (IMP, ATP) in both A549 and BEAS-2B cells (Fig. 5c, d and Supplementary Fig. 8). In addition, compared to the control group, a lower level of ^15^N-labeled ATP was detected in PNP-knockdown cells upon infection (Fig. 4d, Supplementary Fig. 8). As for adenosine salvage, H1N1 enhanced the utilization of ^15^N-labeled adenosine and ^15^N-labeled AXP (ATP, ADP, AMP) production (Fig. 5e, f and Supplementary Fig. 8). Even though the phosphorolytical conversion of adenosine to adenine was not catalyzed by PNP in eukaryotic cells, PNP knockdown still reduced ^15^N-labeled adenosine consumption and AXP production (AMP, ADP, ATP, Fig. 4e, f and Supplementary Fig. 8**)**, which was consistent to the increased level of adenosine in PNP-knockdown A549 and BEAS-2B cells (Fig. 4e, Supplementary Fig. 8). During guanosine salvage, suppressing PNP activity reduced the generation of ^15^N-labeled purine products increased by H1N1 (Fig. 5g, h, and Supplementary Fig. 8). Moreover, the branch of adenosine and guanosine salvage (M + 4) pathway was not significantly affected by H1N1 or PNP (Fig. 5f, h and Supplementary Fig. 8).

Collectively, by metabolite flux analysis, we further demonstrated that, upon infection, PNP inhibition blocked H1N1-enhanced purine salvage while reactivated purine de novo synthesis in alveolar epithelial cells.

### PNP-catalyzed purine nucleosides recycling benefits H1N1 propagation

**Fig. 6:**
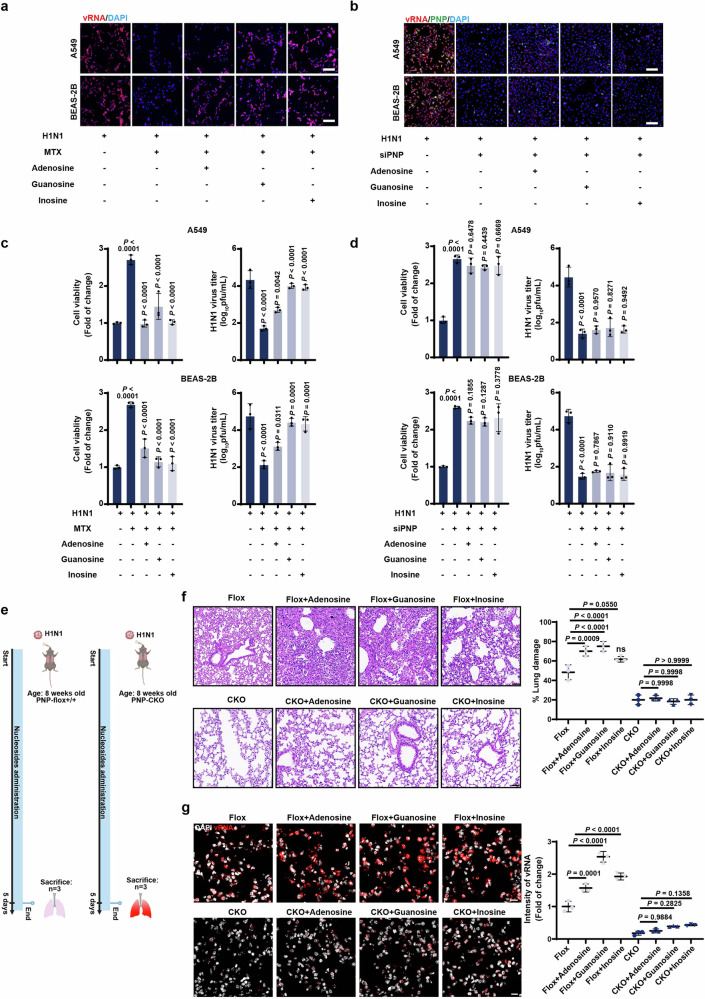
PNP-mediated purine salvage is essential for H1N1 propagation. **a**, **c** The effect of nucleosides on viral replication and cell viability upon MTX treatment in challenged cells. H1N1-challenged A549 and BEAS-2B cells were cultured in medium with 2 μM MTX for 12 h, and then treated by adenosine (50 μM), guanosine (50 μM), or inosine (50 μM) for another 24 h, followed by FISH analysis of vRNA (Scale bar: 100 μm, **a**). Cell viability and viral titer were also examined (**c**). **b**, **d** The effect of nucleosides on viral replication and cell viability was dependent on PNP activity. H1N1-infected A549 and BEAS-2B cells, transfected by NC siRNA or PNP siRNA, were cultured in the addition of 50 μM guanosine, inosine, or adenosine for 24 h. FISH analysis of vRNA, IF assay of PNP, and DAPI staining were performed (Scale bar: 100 μm, (**b**). Cell viability and viral titer were also analyzed (**d**). All the experiments were conducted in triplicates. **e** Diagram of the experimental procedures. Briefly, 8-week-old PNP^flox/flox^ mice and PNP conditional knockout mice were intranasally challenged with 2LD_50_ H1N1. H1N1-infected mice were intragastric administered with adenosine (8 mg/Kg), guanosine (8 mg/Kg), and inosine (8 mg/Kg), respectively. **f**, **g** The inflammatory lesion and viral infection within the lungs of H1N1-infected mice. After 6 days of infection, 3 mice from each group were sacrificed, with the lungs dissociated and subjected to H&E staining (**f**, Scale bar: 50 μm), FISH of vRNA, and IF assay of PNP (**g**). DAPI was used to indicate nucleus (*N* = 6, Scale bar: 10 μm). The area of lung damage and intensity of vRNA (**f**, **g**, the right plots) were shown. All data are presented as mean ± SD; unless otherwise indicated, *N* = 3 biologically independent experiments; Statistical analysis was performed by one-way ANOVA (**c**, **d**, **f**, **g**)

Given that purine nucleotides rewiring in response to H1N1 infection was mediated by host PNP, we employed FISH assays to further verify the PNP-mediated purine salvage in vitro and in vivo. The addition of methotrexate (MTX), a dihydrofolate reductase (DHFR) competitive inhibitor used as an inhibitor for one-carbon metabolism involved in nucleotide synthesis, inhibited vRNA production and diminished viral titer in both A549 and BEAS-2B cells (Fig. 6a, c). This was accompanied by an increase in cell viability (Fig. 6c). Importantly, the inhibition of vRNA and virus production can be reversed by the addition of purine salvage substrate adenosine, guanosine, or inosine (Fig. 6a, c). These results implied that IAV efficiently utilized purine salvage to support viral propagation. However, when PNP was knocked down in infected cells, the addition of extra guanosine or inosine was unable to restore vRNA generation, viral titer, or cell viability, highlighting that the effect of purine salvage on viral replication was relied on PNP (Fig. 6b, d). We further verified the PNP-mediated purine salvage in vivo (Fig. 6e). The administration of the wild-type mice with purine nucleosides after H1N1 infection dramatically aggravated lung damage and intensity of vRNA (Fig. 6f). While PNP-knockout mice significantly suppressed the rapid growth of H1N1 virus and severe lung damage induced by nucleosides supplementation (Fig. 6g).

Taken all the above results together, we have revealed the critical role of host purine salvage in H1N1 replication and host cell viability, demonstrating that the sensitivity of viral infection to purine nucleosides was dependent on PNP activity.

### PNP inhibition exhibits anti-inflammation roles via APRT-AICAR-AMPK axis upon H1N1 infection

Consistent with the effect of H1N1 infection on purine synthesis and the critical role of PNP, a series of purine salvage enzymes were found to be decreased in PNP-knockdown A549 cells, compared to the control group in response to H1N1 infection. However, the de novo purine synthesis enzymes, such as polyfluoroalkyl substances (PFAS), 5-Amino-4-imidazolecarboxamide ribonucleotide transformylase/IMP cyclohydrolase (ATIC), were increased by transcriptomic data (Supplementary Fig. 3a–c). Furthermore, gene set enrichment analysis (GSEA) demonstrated that AMP-activated protein kinase (AMPK) pathway, whose anti-inflammation effect has been extensively studied^40,41^ was activated, while a series of downstream proinflammation signaling (phosphoinositide 3-kinase (PI3K)-Akt, TNF, Janus kinase/signal transducer and activator of transcription (JAK/STAT) and nuclear factor kappa B (NF-κB) pathway) were inhibited (Supplementary Figs. 3d, 4a). To better illuminate the regulatory role of PNP-AMPK axis in influenza-associated inflammation, we further inhibited AMPK phosphorylation by compound C (a well-defined AMPK antagonist) and found that compound C significantly reduced the anti-inflammation response of PNP inhibition, which was evidenced by a much higher level of IL-6 and TNF-α in PNP-knockdown A549 and BEAS-2B cells (Supplementary Figs. 3e, 4b).

Next, we set to uncover the molecular mechanism of PNP-modulated AMPK activation. As discussed in Fig. 4, AICAR, a de novo purine synthesis intermediate, was increased by PNP inhibition. Actually, AICAR was well-known for its activating effect on AMPK.^42^ Moreover, hypoxanthine phosphoribosyltransferase 1 (HPRT1) and adenine phosphoribosyltransferase (APRT), playing central roles in purine salvage and AICAR production,^42,43^ were also found to be upregulated in PNP-knockdown cells (Supplementary Fig. 3c). By knockdown of APRT or HPRT1, we found that APRT, rather than HPRT1, was responsible for PNP-deficiency-induced accumulation of AICAR upon H1N1 infection (Supplementary Fig. 3f). Furthermore, the suppression of APRT inhibited AMPK activation and reversed the expression of proinflammatory cytokines (Supplementary Fig. 3g, 4c). These results indicated that PNP suppression-induced AMPK activation under H1N1 infection was mediated by APRT-catalyzed AICAR accumulation.

Thus, our results unveiled that PNP inhibition activated APRT-AICAR-AMPK signaling to suppress hyperinflammation induced by H1N1 infection, suggesting the hub role of PNP in connecting viral replication and inflammation via purine salvage.

### Purine nucleotides in the blood of IAV-infected patients is correlated with infection and peripheral inflammation

**Fig. 7:**
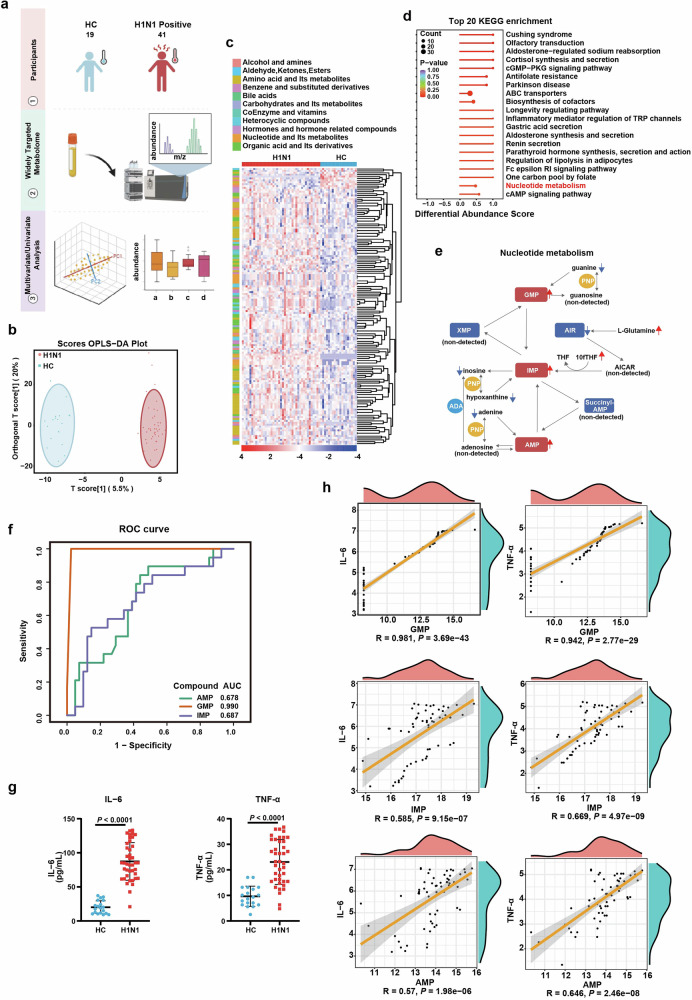
Purine nucleotides in the blood of IAV-infected patients is correlated with infection and peripheral inflammation. **a** The schematic diagram of widely-targeted metabolomic profiling of plasma from H1N1 patients (H1N1 positive) and healthy control (HC). Briefly, the plasma of the peripheral blood from 19 HC and 41 H1N1 patients were subjected to widely-targeted metabolomic analysis. **b** Orthogonal partial least square discriminant analysis (OPLS-DA) for the data from widely targeted metabolomic profiling of HC and H1N1 positive individuals. The HC and H1N1 group were discriminated into two separated clusters by OPLS-DA analysis. **c** Heatmap of identified metabolites. The expression of 657 identified metabolites from the widely-targeted metabolomic data were represented by heatmap. **d** KEGG pathway enrichment analysis of the differentiated expressed metabolites between HC and infected individuals. The top 20 KEGG pathways were enriched, with the annotation of nucleotide metabolism pathway being labeled as red. **e** An overview of purine metabolism pathway alterations and metabolites involved in de novo purine synthesis and purine salvage. Red arrows indicated upregulated metabolites, while blue arrows indicated downregulated ones. **f** AUC-ROC curve analysis of the correlation between purine nucleotide and H1N1 infection. The discriminative capability of purine nucleotide GMP, IMP, and AMP in distinguishing H1N1-infected and uninfected individuals was quantified by area under ROC curve (AUC), with GMP showing the highest AUC. **g** The expression of TNF-ɑ and IL-6 in the peripheral plasma. The concentration of TNF-ɑ and IL-6 of the control and H1N1-infected patients were measured by ELISA. **h** The correlation analysis between inflammatory cytokines and purine nucleotides. The correlation of purine nucleotides (GMP, IMP, AMP) with inflammatory cytokines (TNF-ɑ, IL-6) in the peripheral blood of H1N1 patients. All data are presented as mean ± SD; Statistical analysis was performed by two-tailed Student’s *t*-test (**g**) and Spearman rank correlation coefficient (**h**)

Given the above discussed role of host purine salvage in IAV infection, we further explored the variation of purine metabolism in IAV-infected patients. The peripheral plasma derived from 19 healthy controls and 41 H1N1-infected individuals were subjected to widely-targeted metabolomic analysis. 326 metabolites in positive ion mode and 331 in negative were identified (Fig. 7a). Via orthogonal partial least squares discriminant analysis (OPLS-DA), healthy control (HC) group and IAV-infected group could be distinguished clearly (Fig. 7b and Supplementary Fig. 5a). 146 out of 657 metabolites were significantly associated with IAV infection (*P* < 0.05; Fig. 7c). Consistent with our in vitro/in vivo data, nucleotide metabolism was significantly enriched in H1N1 patients by Kyoto Encyclopedia of Genes and Genomes (KEGG) functional enrichment analysis (Fig. 7d). In particular, all the mononucleotides were significantly increased, implying that H1N1 infection reprogramed host nucleotide metabolism to meet the massive demand for viral replication (Supplementary Fig. 5b). Similar to the alteration pattern revealed in IAV-infected AECs, in H1N1-infected patients, glutamine of de novo synthesis was also accumulated, while metabolites of purine salvage including all the purine bases (adenine, hypoxanthine, guanine) and inosine were decreased (Fig. 7e).

Next, we generated ROC curves to assess if the nucleotide metabolites signatures could be used as diagnostic biomarkers for H1N1. The ROC curves revealed that GMP, with the AUC value of 0.990, was the most efficient metabolite in discriminating H1N1 positive patients from uninfected controls (Fig. 7f). The concentration of IL-6 and TNF-ɑ in the peripheral blood were also dramatically increased in patients with H1N1 (Fig. 7g). Moreover, the levels of GMP, IMP and AMP were positively correlated with the peripheral expression of IL-6 and TNF-ɑ (Fig. 7h). To further investigate the relationship between the biomarkers and disease outcomes, we stratified the enrolled patients into mild and severe groups according to their clinical characteristics(Supplementary Table 5).^44^ Subsequently, we re-evaluated the correlation between the biomarkers (GMP, AMP, and IMP) and clinical outcomes. The results indicated that GMP was positively correlated with the severity of the disease, suggesting that GMP may serve as a potential biomarker for disease progression (Supplementary Fig. 5c, d).

Overall, these observations validated the transition of purine metabolism from de novo to salvage pathway in H1N1-infected patients, and provided evidence for the potential biomarker role of nucleotide metabolite in disease diagnosis.

### PNP inhibitors are promising host-targeted anti-influenza drugs

**Fig. 8:**
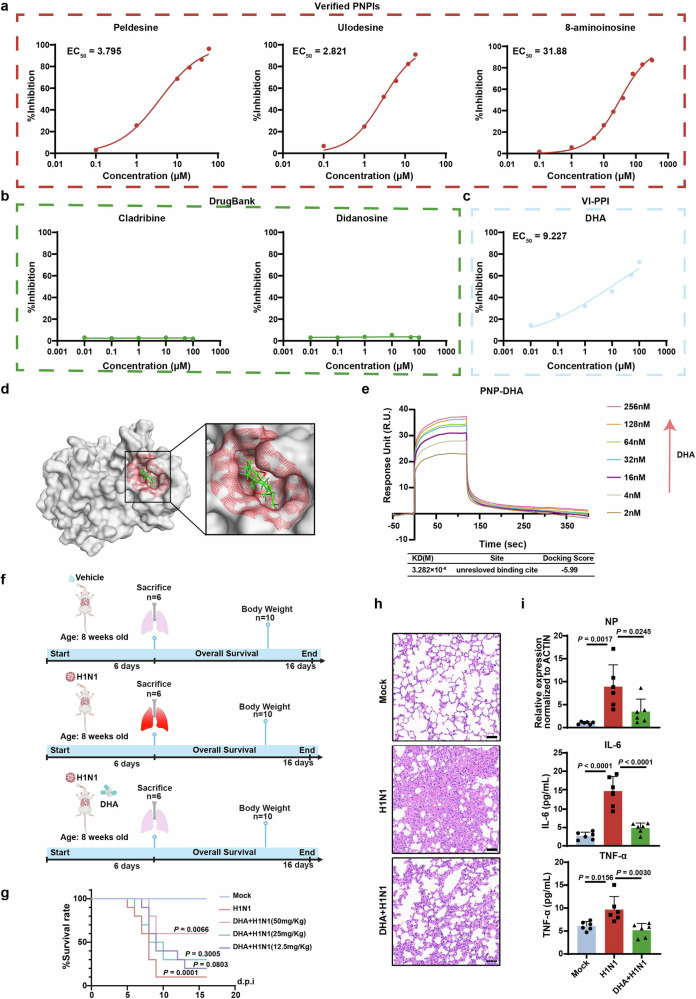
VI-PPI-predicted dihydroartemisinin (DHA) plays an anti-influenza role in vitro/in vivo by targeting host PNP. **a** The anti-H1N1 activities of the verified PNPIs. MDCK cells infected with H1N1 at an MOI of 0.01 were treated with PNPIs (peldesine, ulodesine, ganciclovir) at different doses for 24 h, followed by analysis of the viral copy number in the supernatant of H1N1-infected cells. **b** The effect of DrugBank-predicted PNPIs on H1N1 replication. MDCK cells infected with H1N1 at an MOI of 0.01 were treated with cladribine or didanosine at different concentrations for 24 h, followed by analysis of the viral copy number in the supernatant of H1N1-infected cells. **c** The anti-H1N1 activity of VI-PPI-predicted PNPI. H1N1-infected MDCK cells were treated with different doses of DHA, which was predicted by VI-PPI network, and then the viral copy number in the supernatant was analyzed. **d** Molecular operating environment (MOE) analysis of the interaction between PNP and DHA. The potential binding sites in PNP are highlighted in red and DHA is indicated in green. **e** The kinetic profile of DHA-PNP binding reaction by surface plasmon resonance (SPR) assay. PNP was immobilized to the surface of the sensor chip. The binding and dissociation of DHA at different concentrations with PNP chip were monitored. The predicted docking score and affinity parameters of DHA binding to PNP are displayed in the lower chart. (**f–i**) The in vivo effect of DHA on H1N1 infection. **f** The schematic of the in vivo assay of the effect of DHA on H1N1-infected mice models. Briefly, mice were randomized into 3 groups (mock, H1N1 plus vehicle (90% corn oil and 10% DMSO), H1N1 plus DHA (12.5 mg/Kg, 25 mg/Kg, 50 mg/Kg)). Vehicle or DHA were intraperitoneally administered for 6 consecutive days. **g** The survival rate was monitored for 16 consecutive days after infection (*N* = 10). **h** The lung tissue of the mice was dissected for H&E analysis 6 days after vehicle or DHA treatment (*N* = 6, Scale bar: 50 μm). **i** Expression level of nuclear protein (NP) of H1N1 in mice lung (*N* = 6) measured by qRT-PCR on day 6 post infection. The concentration of TNF-ɑ and IL-6 levels in mice plasma (*N* = 6) was measured by ELISA on day 6 post infection. All data are presented as mean ± SD; unless otherwise indicated, *N* = 3 biologically independent experiments; Statistical analysis was performed by Log-rank test (**g**) and one-way ANOVA (**i**)

Multiple PNP inhibitors (PNPIs) have been developed, exhibiting potential therapeutic effects in clinical. Peldesine is a PNPI for treatment of autoimmune disorders and has entered clinical trials for human immunodeficiency virus (HIV) infections. Ulodesine is an investigational PNPI used to treat hyperuricemia and gout.^45^ 8-aminoinosine is reported to exert diuretic and natriuretic activity by inhibiting purine nucleoside phosphorylase.^46^ All the three PNPI showed marked inhibitory effect on the viral copy number in the supernatant of H1N1-infected MDCK cells, indicating the anti-influenza potential of PNPIs (Fig. 8a).

However, there is no FDA-approved PNPIs available to date. As discussed before, that VI-PPI network incorporated drug targets data, we tried to screen potential PNP-targeting drugs (FDA-approved) via this newly created model. The top 200 PNP-related genes were extracted using Random Walk with Restart algorithm (RWR), followed by an enrichment analysis based on the approved drug targets derived from DrugBank database. As a result, dihydroartemisinin (DHA, also known as artenimol), ranked the first place with the lowest p value and the most overlapped genes between PNP-related molecules and drug targets, was capable of inhibiting H1N1 replication (Fig. 8c and Supplementary Fig. 6a). Moreover, DHA increased the viability of infected A549 and BEAS-2B cells in a dose-dependent manner, peaking at the concentration of 1 µM. And thus, 1 µM DHA was used afterwards. The viral load in AECs was also markedly inhibited by DHA at 1 µM (Supplementary Fig. 6b). Additionally, cladribine (deoxyadenosine analog) and didanosine (deoxynucleoside analog), suggested by the DrugBank database to be capable of interacting with PNP, were also evaluated for their effect on H1N1-infected cells. However, no significant protective roles were found in cells treated with these two drugs (Fig. 8b), indicating that VI-PPI-predicted DHA might be a novel PNP-targeting compound in IAV treatment.

To demonstrate if there was a direct inhibition of DHA on PNP, we evaluated the protein level of PNP upon DHA treatment, and found that IAV-induced upregulation of PNP in A549 and BEAS-2B cells were reduced by DHA (Supplementary Fig. 6c). Additionally, overexpression of PNP significantly reversed DHA-induced increase of cell viability and reduction of H1N1 viral titer, indicating that the anti-viral effect of DHA was mainly mediated by its effect on PNP (Supplementary Fig. 6d). Then, molecular docking analysis predicted a docking score of −5.99 to imply a promising binding between DHA and PNP. Also, a potential pocket consisting of 14 amino acids within PNP were identified, and two H-bonds were implied to be responsible for the binding (Fig. 8d and Supplementary Fig. 6e). Importantly, surface plasmon resonance (SPR) assay confirmed the direct interaction between DHA and PNP, showing a dissociation equilibrium constant (KD) of $3.282\times 10-8\,$M (Fig. 8e).

Subsequently, the anti-influenza effect of DHA was examined in vivo. H1N1-challenged mice were intraperitoneally administered with DHA or vehicle (90% corn oil and 10% DMSO) daily for a consecutive six days (Fig. 8f). DHA induced a robust extended survival rate and an accelerated body weight restoration upon IAV when compared to vehicle-treated group (Fig. 8g and Supplementary Fig. 6f), with PNP downregulation and AMPK activation observed in the lungs (Supplementary Fig. 6g). DHA administration (50 mg/Kg/day) also significantly mitigated the pulmonary pathological changes of the infected mice (Fig. 8h), reducing leukocytes infiltration, proinflammatory cytokines production (IL-6, TNF-ɑ), and viral nucleoprotein (NP) expression (Fig. 8i).

Collectively, our results clearly demonstrated the potential of PNP as an anti-influenza host target, with PNP inhibitors being efficient to combat IAV. More importantly, DHA, predicted by VI-PPI as a novel PNP-interacting molecule, could directly bind PNP to constrain viral replication and host hyperinflammation.

## Discussion

The dynamic viral-host interactions drive the occurrence and progression of IAV infection and are critical to determine the outcome of the disease. Host genes, exhibiting hub roles in linking viral infection and host inflammation, represent a repertoire of highly promising candidates for antiviral target screening. Here, by creating VI-PPI network focusing on the host hub genes driving IAV progression, we identified PNP, a vital enzyme in purine salvage, as a potential anti-viral target. The main findings of our study were: (1) PB1-F2 was revealed as the first viral transcription factor of H1N1 to activate host PNP; (2) the knockdown of PNP elicited a metabolism shift from H1N1-enhanced purine salvage to de novo pathway to reduce viral replication in AECs; (3) PNP suppression downregulated the inflammation level of infected cells through the activation of APRT-AICAR-AMPK axis; (4) the increased level of purine salvage was also confirmed in H1N1 patients, with the peripheral nucleotide metabolite exhibiting potential diagnostic roles; (5) DHA, predicted by VI-PPI as a novel PNP-targeting drug, was proved to bind PNP directly to play a protective role against IAV in vivo. It is for the first time that host PNP was revealed to play hub roles within viral-host interaction to modulate IAV replication and inflammation via purine salvage.

PPI-based screening has long been considered as a powerful tool for gene discovery, focusing on molecules with more interacting neighbors. While, the so-called hub genes that link the multiple steps during the dynamic changing of the disease are usually ignored for their less neighbors. Here, we constructed an anti-viral target screening model to reveal the inner correlation between the initial IAV infection and subsequent severe pneumonia, which could be described as “migration” within VI-PPI network. Moreover, we found that the pneumonia-related genes were somehow different to the factors that interacted with H1N1 viral proteins, indicating again that some key genes were located within the “migration” paths from the start (H1N1 infection) to the end (pneumonia) on the network. Through the analysis of *local betweenness centrality*, the hub genes of VI-PPI network were better extracted, identifying PNP as a potential anti-IAV target for the first time.

PNP is a crucial enzyme in purine salvage and responsible for the phosphorylation of inosine and deoxyinosine into hypoxanthine, as well as guanosine and deoxyguanosine into guanine, which prompted us to unveil the role of purine salvage during influenza. Unlike de novo purine pathway, the role of purine salvage in viral infection has not received enough attention, in part because the methods used for measuring nucleotide salvage are less well established. Very recently, the critical effects of nucleotide salvage pathway have been unveiled on picornaviruses infection, the resistance of intestinal cells of C. elegans to pathogens, and *Haemophilus influenzae* infection.^20,21,47^ However, no data have been published about the alterations and effects of purine salvage in influenza. We found that PNP was significantly upregulated upon H1N1 infection, accompanied by an increased consumption of purine nucleosides (inosine, guanosine, adenosine) and an accumulation of de novo substrate glutamine. Nucleoside derivatives have been suggested as a promising anti-viral therapeutic strategy by interfering viral hijack of host nucleotide metabolism.^48^ Similarly, in our study, inhibition of PNP was efficient to induce a metabolic pattern shift from rapid purine salvage to rate-limiting de novo synthesis in AEC, resulting in viral replication downregulation and cell viability upregulation. Moreover, upon H1N1 challenge, AEC-specific PNP conditional knockout mice (Nkx2-1-CreERT2, PNP^flox/flox^) exhibited a higher survival rate and an accelerated body weight restoration, as well as reduced inflammatory pathological injuries in the lungs, better elucidating the critical role of AEC PNP in influenza. Of note, we found that glutamine was markedly increased upon H1N1 infection. An increased level of glutamine has been revealed to support the infection of cytomegalovirus and syncytial virus.^49,50^ Therefore, the enhanced H1N1 replication might be determined by both PNP-involved purine salvage and the accumulation of glutamine resulted from reduced de novo synthesis. However, glutamine has also been reported to exert antiviral roles. Glutamine could induce a marked increase in interferon-stimulated genes (ISGs) expression and interferon-ß signaling through oxidative phosphorylation (OXPHOS)-dependent manner in A549 cells upon dengue virus and Zika virus infection.^51^ Thus, the outcome of viral infection was contributed by the balance between the pro- and anti-viral role of glutamine. However, in this study, we focused primarily on the effects of PNP-mediated purine salvage pathway but did not explore the impact of glutamine or other alternative metabolic pathways during H1N1 infection. Moreover, an investigation into the role of the interferon response was lacked. These limitations restrict the comprehensiveness of our findings and will be addressed in future research.

Furthermore, viral PB1-F2 was revealed as a vTR for PNP. Viral transcriptional regulators, encoded by viral genome, are vital to viral-host interaction, as vTRs could alter the expression of not only viral genes but also host genes. However, the current exploration of vTRs is only the tip of the iceberg, with only 419 vTRs belonging to 20 different virus families being reported,^32^ among which no vTR of influenza has ever been reported. Via establishing A549 cells stably expressing various H1N1 proteins (HA, NA, NP, PA, PB1, PB1-F2, PB2, NS1, M1, M2), PB1-F2 was revealed to be the most potent viral protein to increase the expression of PNP. Moreover, PB1-F2 protein of other strains of IAV (H5N1, H7N7, H3N2) was also capable of upregulating PNP, indicating that PB1-F2-induced PNP upregulation was universal in IAV infection. PB1-F2 functions as the core component of the viral polymerase to contribute to the pathogenesis and comorbidity of IAV.^52,53^ It could induce mitochondrial fission or mitophagy to attenuate the innate immune response and compromise IFN synthesis.^54^ More importantly, the genetic and functional diversities of PB1-F2 protein make it closely associated with the viral replication and virulence for various strains of influenza A virus.^55^ In this study, PB1-F2 was revealed to significantly increased the luciferase activity of the reporter gene for PNP-promoter. Based on Alphafold 3^33^ and DeepPBS,^34^ the binding motif of PB1-F2-PNP promoter complex was predicted and the interaction of PB1-F2 to the TATA-box of PNP promoter further verified.

The variation of purine metabolism during IAV infection and its correlation with peripheral inflammation were also verified in IAV-infected patients. Clinically, disordered metabolic signatures with metabolite accumulation or deficiency have been proposed as potential biomarkers for the diagnosis and prognosis of diseases.^56,57^ However, no such metabolite biomarker has been revealed in H1N1 infection. Purine metabolism was significantly enhanced in H1N1 patients. The levels of GMP, IMP and AMP were positively correlated with the disease severity and the peripheral level of IL-6 and TNF-ɑ, with GMP exhibiting the most relevance, which indicated the potential of nucleotide metabolites in diagnosing influenza-associated pathologies.

More importantly, the hub role of PNP in connecting viral replication, host metabolism, and inflammation was revealed by our data. Controversial effects of PNP-mediated purine metabolism on immune system have been discussed by other groups. PNP deficiency induces T-cell depletion and severe immunodeficiency via the accumulation of dGTP, which has been used as a therapeutic strategy for autoimmune diseases.^24,45^ Meanwhile, pharmacologic PNP inhibitors are able to enhance innate immune response as IFN release, NK cell activation, and dendritic cell maturation through aggregation of guanosine, leading to increased capability of anti-tumor and anti-bacteria effect and immune responses to HBV vaccines.^58^ In our study, suppressing PNP activity enhanced the accumulation of de novo intermediate AICAR, which was a highly selective and potent activator for AMPK. It was reported that AICAR-boosting AMPK-TBK1 cascade could improve anti-viral immunity in multiple animal models.^59^ We also noticed that PNP inhibition was accompanied by an increase of APRT expression, which has been reported to be capable of catalyzing AICAR synthesis.^43^ Based on our data, PNP inhibition significantly induced accumulation of adenosine upon H1N1 infection, which would probably promote compensatory upregulation of salvage pathway involving ADA and APRT to enhance nucleotide production to fuel H1N1 infection. Taken together, we found that PNP-inhibition ignited APRT-AICAR-AMPK axis to attenuate a series of downstream proinflammation signaling pathways. Therefore, our study revealed the bridging role of PNP and PNP-mediated purine salvage in influenza, and the anti-influenza effect of PNP deficiency was contributed by its dual inhibition on viral replication and host inflammation.

However, there is still no Food and Drug Administration (FDA)- or European Medicine Agency (EMA)-approved PNP-targeting therapeutics available. By taking the advantage of VI-PPI network, we identified dihydroartemisinin (DHA, an artemisnin derivatives extracted from Chinese medicine *Artemisia annua L* used in the treatment of uncomplicated *Plasmodium falciparum* infections) as a natural PNP inhibitor. More importantly, the direct binding between PNP and dihydroartemisinin was demonstrated by SPR analysis. In addition, the inhibition of PNP by DHA and the protective role of DHA against H1N1 were validated in both in vivo and in vitro models. DHA has also been reported to have anti-inflammatory activities by other groups. Muramidase-released protein (MRP) -induced innate inflammation was revealed to be ameliorated by DHA through inactivation of TLR4-dependent NF-κB signaling.^60^ Moreover, DHA was potent in reducing inflammatory cell infiltration and suppressing the production of pro-inflammatory cytokines.^61,62^ In this study, for the first time, the anti-inflammation effect of DHA was linked to purine salvage by its interaction with PNP, which deepened understanding of DHA in modulating metabolism and infection.

Taken together, via VI-PPI inflammatory network, we identified PNP as a vital nexus molecule during IAV infection, revealing its bridging role in connecting viral replication, purine synthesis adaptation, and host inflammation for the first time. Furthermore, DHA, predicted by VI-PPI network as well, was proved to bind to PNP directly, with its protective role against H1N1 infection demonstrated in vivo. Our study sheds new light on a “two-for-one” strategy by targeting purine salvage in combating IAV-related pathology, suggesting PNP as a potential novel anti-influenza host target.

## Materials and methods

### In vitro studies

A549, BEAS-2B and MDCK were purchased from ATCC. Cells were incubated at 37°C in 5% CO_2_. When the cells reached 80% confluent, they were split for experiment. All cell lines were not used for more than 10 passages. A549 and BEAS-2B cells were cultured in DMEM/F12 (Corning) supplemented with 10% fetal bovine serum and 1% penicillin/streptomycin (Corning). MDCK cells were cultured in DMEM (Corning) supplemented with 10% fetal bovine serum and 1% penicillin/streptomycin (Corning).

H1N1 virus (Puerto Rico/8/1934, PR8), H3N2 (Swizerland/9715293/2013), and H5N1 (Wuhan/EZ02/2016) was propagated in embryonated chicken eggs (9-day-old), and then collected and measured with hemagglutination activity in a BSL-3 laboratory.

### Animal studies

All animal experiments were approved by the Animal Research of Committee of AMMS and performed following the National Institutes of Health Guidelines on the care and Use of Animals (IACUC-IME-2024-009). The BALB/C mice were acquired from Sipeifu Biotechnology (Beijing, China). Nkx2-1-CreERT2 and PNP^flox/flox^ mice were obtained from Shanghai Model Organism Center. To generate AEC PNP-deficient mice, the Nkx2-1-CreERT2 mice were crossed with PNP^flox/flox^ mice to obtain Nkx2-1-CreERT2^+^- PNP^flox/flox^ (PNP CKO). Nkx2-1-CreERT2^-^- PNP^flox/flox^ mice from littermates were as controls. Mice were housed in a specific pathogen-free (SPF) environment. Only adult male mice were used in our study to avoid the impact of gender differences. All animal experiments were assessed according to concealed allocation and blinding of outcome.

### Clinical study participants and data collection

A total of 41 patients with H1N1 were retrospectively recruited from September 1 to October 30, 2023 at Sixth Medical Center of PLA General Hospital. All enrolled patients were confirmed to be positive for H1N1 nucleic acid by RT-PCR. Blood samples from 19 healthy controls were collected from individuals who had annual physical examination during sample collection period (Table S4). The admission data of these patients were collected and checked independently by two physicians. The study was performed in accordance with the Declaration of Helsinki principle for ethical research. The study protocol was approved by the ethics committee of the Sixth Medical Center of PLA General Hospital (HZKY-PJ-2024-30).

### Methods details

#### VI-PPI network construction

VI-PPI is a protein-protein interaction network consisting of genes associated with viral infection and inflammation. Nodes in VI-PPI were connected according to the internal PPIs information.

The inflammation-associated genes (IAGs): We extracted the IAGs from following four primary approaches: (1) We compiled a comprehensive list of 107 inflammation-related processes from PathCards^63^ and included the genes associated with these pathways. (2) 212 diseases related to the Gene Ontology item “acute inflammatory response” (GO:0002526) and Gene-disease association were collected from CTD database.^64^ A hierarchical clustering algorithm was used for diseases deduplication, and the genes associated with at least 3 of these diseases were collected. (3) genes listed in “Inflammation” and “Inflammation_II” panels from biomarker assay list provided by Olink platform (https://olink.com/products-services/explore/) were included; (4) genes listed under the GO item “inflammatory response” (GO:0006954) were collected.

The viral infection-associated genes (VIAGs): We compiled human genes interacting with viral proteins from HVIDB,^65^ which integrated several PPI data sources, including HPIDB,^66^ BioGRID,^67^ VirHostNet,^68^ and PHISTO.^69^ Additionally, disease genes involved in common viral infections were collected from MalaCards^70^ and GeneCards, the top 200 genes related were included.

The drug-target genes (DTGs): We extracted druggable human genes based on approved drug-target links provided by DrugBank.^71^

The signaling pathway gene (SPGs): In order to analyze the signaling pathway involved in the process of inflammation caused by viral infection, we extracted the genes under 45 common signaling pathways from KEGG database.^72^

PPI data: We collected the human PPIs from four databases: IntAct,^73^ MINT,^74^ BioGRID,^67^ and STRING.^75^ To ensure the quality of the data, only the physical interactions were retained, and the following interactions were excluded: (1) PPI pairs without experimental validation; (2) PPI pairs with low confidence (combined score < 0.7) from STRING database; (3) For IntAct and MINT, PPI pairs with MIscore lower than 0.485 were excluded, according to the threshold provided by previous study.^76^ Each protein was converted to gene symbol using the ID mapping tool provided by Uniprot. Finally, the PPI pairs were used to link the aforementioned four types of genes. The largest connected component of this network was named VI-PPI and subjected to further analysis.

#### Network visualization and topological properties analysis

All the networks involved in this study were subsequently visualized using Cytoscape (version 3.9.1).^77^ Network properties were calculated using igraph package (version 1.4.1) in R.

#### SAFE analysis

We applied a clustering algorithm SAFE v1.0^78^ in Cytoscape to determine tightly interconnected functional modules in the VI-PPI network. The network layouts were generated using the edge-weighted spring embedded layout. SAFE analysis was run using the default parameters.

#### Identification of differential expression gene

Differential expression genes (DEGs) were identified from the transcriptome expression profile by the R package DESeq2 (version 1.39.4).^79^ Genes with |log_2_(Fold Change)| > 2 and FDR < 0.01 were deemed as DEGs.

#### GO annotation and enrichment analysis

GO annotation, GSEA, and KEGG pathway enrichment were conducted and visualized using clusterProfiler R package (version 4.6.2).^80^ Terms with an FDR adjust *P* < 0.05 were deemed statistically significant.

#### Identification of key genes for viral infection

Viral products of H1N1 induce a pro-inflammatory response, but that in excess induces severe pneumonia.^81^ We collected the human genes which could interact with H1N1 as H1N1-related genes from HVIDB,^65^ as well as the pneumonia-related genes from CTD.^64^ According to the control theory of network,^82^ the genes connected the H1N1-related genes and pneumonia-related genes were regarded as key genes. Thus, we used *local betweenness centrality* to measure the bridge role of gene *v* for connecting the given two gene lists *S* and *T*:

$${local\; betweenness}=\frac{1}{{|S||T|}}\sum _{s\in S,\,t\epsilon T}\frac{{\sigma }_{{st}}(v)}{{\sigma }_{{st}}}$$

where *σ*_*st*_ is the total number of shortest paths from node s to node t and *σ*_*st*_ (v) is the number of those paths that pass through *v*.

We randomly selected 100,00 gene lists with same size as pneumonia-related genes, and calculated the value of *local betweenness centrality* value for each involved gene between H1N1-related genes and these sampled lists. Each involved gene will get a reference distribution. Gene *v* which satisfiedlocal betweenness> Q3+3*(Q3-Q1) was regarded as gene at important position, where *Q1* and *Q3* are the 25th and 75th percentile of the reference distribution.

We used *local betweenness centrality* to identify the genes which played important bridge roles between H1N1-related genes and pneumonia-related genes. The same procedure was applied for H1N1, H3N2, H5N1, and H7N7. The common genes of these four viruses were collected for further analysis.

#### Prediction of protein-DNA binding site

Alphafold 3^33^ was utilized to predict protein-DNA complex structure based on the protein and DNA sequence. For motif prediction, the predicted protein-DNA complex was input into DeepPBS^34^ to obtain the specific binding site.

#### Virtual screening for candidate compounds

Random Walk with Restart (RWR) algorithm was utilized to identify genes which had a close connection with PNP on VI-PPI. Drug-target information was extracted from DrugBank database.^71^ An enrichment analysis was performed on top 200 genes ranked by RWR scores, with a p-adjust value threshold set to 0.05. Molecular Operating Environment (MOE) software was used for compound-protein docking and visualization.

#### siRNA knockdown and plasmid overexpression

For PNP knockdown, PNP expression in A549 or BEAS-2B cells was knocked down using siRNA. Briefly, Lipofectamine RNAiMAX (Invitrogen) and PNP siRNA (Genechem) were mixed for 5 min and added to cells following the recommended instruction. For PNP overexpression, X-tremeGENE HP DNA (Roche) and PNP plasmid (Genechem) were mixed for 15 min and added to cells following the manufacturer’s instructions. For stable IAV-protein overexpressed A549, HA, NA, NP, PA, PB1, PB1-F2, PB2, NS1, M1, M2 amplified products were inserted into lentivirus vector PLVX. The virus supernatant was collected after 48 h and 72 h to obtain the IAV-genes overexpressed lentivirus PLVX-Teton-Puro-T2A-CopGFP. IAV-gene lentivirus were added into A549 cells for 24 h, respectively. The transfected cells were screened and cultured with 1% puromycin for 1 week. The expression efficiency of IAV-gene was observed under fluorescence microscope. After selection, cells were treated with tetracycline to express IAV-genes.

#### Plaque Assay

To evaluate the anti-IAV activity of PNP knockdown and DHA, MDCK cells were seeded in 6-well plates and cultured with supernatant from IAV-infected A549/BEAS-2B cells with or without treatment of PNP knockdown or DHA for 1 h at 37 °C. An agar overlay containing 2% Oxoid agar, 0.2% BSA, DEAE dextran, 2 mg/ml TPCK and DMEM/F12 was applied to the wells. After 48 h, the cells were fixed and stained with crystal violet to visualize plaques.

#### Live cell number quantification assay

Cell viability was determined by Cell Counting Kit-8 (CCK-8) assay, 5 × 10^3^ cells/well of A549/BEAS-2B in 96-well plates (Corning) were infected with 10 µL virus with solvents. Then, cells were treated with different concentrations of antiviral candidates for 24 h, and cell viability was measured by CCK-8 assay (Vazyme). The optical density (OD) values were obtained from Microplate Reader (Infinite M PLEX, Tecan). The fold change of cell viability was calculated dividing the OD of H1N1-infected cells by the OD of mock infected cells with the same treatment.

#### Cytokine quantification

In vitro: levels of TNF-ɑ and IL-6 were quantified in supernatants of PNP knockdown applying commercially available Human ELISA kits (MULTI SCIENCES) following the manufacturer’s instructions. In vivo: mice from mock, IAV-infected group and DHA treated group were sacrificed at 6 dpi and serum was collected for TNF-ɑ and IL-6 quantification using Mouse ELISA Kit (MULTI SCIENCES) under the manufacturer’s instructions. Patients’ plasma: TNF-ɑ and IL-6 quantification in patients’ plasma were carried out using Human ELISA kits (MULTI SCIENCES) under the manufacturer’s instructions.

#### Predictions of Binding Sites for PB1-F2

We used the web-based version of AlphaFold 3 to predict the structure of the protein-DNA complex, employing the default parameters of the multimer model. The web interface can be accessed via the provided link: https://deepmind.google/technologies/alphafold/alphafold-server/. The predicted structure was then processed using the DeepPBS model, a geometric deep-learning model designed to predict binding specificity from experimental or predicted protein-DNA structures. The installation and usage pipeline for DeepPBS can be accessed via the GitHub repository: https://github.com/timkartar/DeepPBS. To ensure the accuracy of the predictions, we utilized the default parameters provided by DeepPBS.

#### Luciferase reporter assay

To examine the effect of PB1-F2 on PNP expression, the luciferase reporter plasmid containing the full-length PNP (pGL3-PNP-promoter) was constructed and co-transfected with PB1-F2-expressing plasmid and Renilla luciferase reporter into 293T cells. After 48 h, the luciferase activity of the reporters was examined by the Dual Luciferase Reporter Assay kit (Promega) according to manufacturer instructions.

To confirm the binding core within PNP promoter for PB1-F2, the luciferase reporter plasmids containing PB1-F2-binding sequence (−50 ~ + 1 upstream of TSS) of PNP promoter with or without TATA-box mutation were constructed. Each plasmid was co-transfected with PB1-F2-expressing plasmid and Renilla luciferase reporter into 293T cells for 48 h, followed by Dual Luciferase Reporter Assay.

#### ChIP-PCR

ChIP followed by PCR analysis was performed on 293T cells to evaluate PB1-F2 binding in the promoter region (TATA-box) of PNP. The PB1-F2-3Flag plasmid was constructed and transfected into 293T cells. After 48 h, Rabbit polyclonal antibody to Flag or its respective IgG isotype control was used for ChIP. Primers for amplification of the regions with PB1-F2 binding sequence in the promoter of PNP was included in Table S3.

#### Targeted intracellular metabolites profiling

At 24 hpi, cells were washed with room temperature PBS after media was removed. A549 cells were incubated at −80 °C for 30 min, harvested with cell scraper, and centrifuged (21,000 × *g*, 5 min) to precipitate proteins. The supernatant was collected and dried down in a vacuum centrifuge at 4 °C. For LC-MS analysis, the samples were resuspended in 100 µL acetonitrile/water solvent and centrifuged (14,000 × *g*, 15 min) at 4 °C. Metabolite profiling was performed using an UHPLC (1290 Infinity LC) coupled to a QTRAP MS (6500+). For RPLC separation, the column temperature was set at 40 °C, and the injection volume was 2 µL. Mobile phase A: 5 mM ammonium acetate in water, mobile phase B: 99.5% acetonitrile A gradient (5% B at 0 min, 60% B at 5 min, 100% B at 11–13 min, 5% B at 13.1–16 min) was then initiated at a flow rate of 400 μL/min, The sample was placed at 4 °C during the whole analysis process. 6500 + QTRAP (AB SCIEX) was performed in positive and negative switch mode. The ESI positive source conditions were as follows: Source temperature: 580 °C; Ion Source Gas l (GS1): 45lon Source Gas 2 (GS2): 60; Curtain Gas (CUR): 35, Ion Spray Voltage (IS): +4500 V; The ESI negative source conditions were as follows: Source temperature: 580°C, Ion Source Gas l (GS1): 45; Ion Source Gas 2 (GS2): 60; Curtain gas (CUR): 35; Ion Spray Voltage (IS): −4500 V. MRM method was used for mass spectrometry quantitative data acquisition. A polled quality control (QC) samples were set in the sample queue to evaluate the stability and repeatability of the system.

#### Purine metabolic flux assays

To determine the de novo purine synthesis flux in A549 cells, ^15^N-amide-glutamine strategies were used.^83^ A549 cells seeded in biological triplicate 10 cm^2^ dishes were cultured in glutamine-free DMEM (Thermo Scientific) containing 10% dialyzed FBS and labeled with 4 mM ^15^N-amide-glutamine (Cambridge isotope laboratories) for 4 h. For purine salvage flux in A549 cells, cells were cultured nucleosides-free MEM ɑ (Gibco) containing 10% dialyzed FBS and labeled with 20 μM U-^15^N-adenosine (Cambridge isotope laboratories), 20 μM U-^15^N-inosine (MedChemExpress), or 20 μM U-^15^N-Guanosine (MedChemExpress) separately for 4 h. After labeling, cell samples were treated with a specific mixture of solvents (methanol:acetonitrile:water = 4:4:2), subjecting it to an ice bath and ultrasonication for 15 min, then chilling at −40 °C for 20 min, followed by centrifugation at 15,000 × *g* for 15 min at 4 °C. After this, 1 mL of the supernatant was taken and dried under warm conditions with nitrogen. Finally, the dried material was resuspended in 50 μL of a 50% acetonitrile-water solution (1:1 volume ratio) for UHPLC-HRMS analysis. Chromatographic separation was performed on a Vanquish UHPLC system with a ACQUITY UPLC BEH Amide (2.1 × 100 mm, 1.7 μm). The injection volume was 2 μL and the flow rate was 0.4 mL/min. The mobile phases consisted of (phase A) water with 15 mM ammonium acetate and (phase B) acetonitrile with 15 mM ammonium. A linear gradient elution was performed with the following program: 0–0.5 min, B:95%; 0.5-7 min, B:95-65%; 7-8 min, B:65–50%; 8–9 min, B:50%; 9–9.1 min, B:50-95%; 9.1–12 min, B: 95%. The eluents were analyzed on a ThermoFisher QE HF-X in Heated Electrospray Ionization. The spray voltage was set to 2.5 KV. Capillary and Probe Heater Temperature were separately 325 °C and 300 °C. Sheath gas flow rate was 30, and Aux gas flow rate was 10. S-Lens RF Level was 50. Full scanning was operated at a high-resolution of 60000 FWHM at a range of 70–1050 m/z.

#### FISH and IF

A set of SWEAMI FISH probes, targeting H1N1 genomic RNA, was obtained from Servicebio Technology. Expression of PNP protein was detected by using an anti-PNP antibody (H-7) purchased from Santa Cruz Biotechnology. Cells grown on chambered dishes were fixed with 4% PFA for 1 h buffered with PBS overnight in the BSL2 laboratory. Samples were then permeabilized with 70% ethanol and subjected to IF and FISH following the standard manual. MultiQuant or Analyst was used for quantitative data processing. The OCs were processed together with the biological samples. Metabolites in OCs with coefficient of variation (CV) less than 30% were denoted as reproducible measurements. After sum-normalization, the processed data were uploaded into before importing into SIMCA-P (version 14.1, Umetrics), where it was subjected to OPLS-DA. Significance was determined using an unpaired Student’s *t*-test, *P* < 0.05 was considered as statistically significant.

#### Bulk RNA-seq

A549 cells were transfected with NC or siPNP and then infected by H1N1 at MOI = 5 for 24 h. Total RNA from two groups were extracted using Trizol Reagent (ThermoFisher). 1–3 µg of each sample were used for library preparation. Each sample was performed in triplicate. Libraries were indexed, pool and sequenced on MGI platform and 150 bp paired-end reads were generated. Raw RNA-seq data were processed through Data Quality Control using FastQC Tool and clean reads were obtained by removing low-quality reads, reads with adapters and reads containing undetermined base information. HISAT2 v2.0.5 was used for generating the index of the reference genome. Finally, DESeq2 provided statistical analysis for identifying differentially expressed genes. Genes with *P* < 0.05 and |log_2_(Fold change)| > 2 were assigned as differentially expressed genes.

#### ScRNA-seq data source and processing

H1N1-challenged scRNA-seq dataset was obtained from our previous study,^27^ which was uploaded on the Genome Sequence Archive in National Genomics Data Center, China National Center for Bioinformation that are publicly accessible at https://ngdc.cncb.ac.cn/gsa (GSA: CRA013575).^84^ Analyzes were performed under R (v.4.3.2) with Seurat (v4.3.0).

#### Surface plasmon resonance (SPR)

To study PNP’s binding to DHA, SPR with a Biacore T200 and CM7 chip was used for coupling measurement. Human PNP (TOPSCIENCE) was attached to CM7 chip by Amino action. Peldesine (BCX 34) dihydrochloride, a reported PNP inhibitor,^85^ was employed as positive control. The assay used DHA at concentrations from 2 to 256 nM in PBS-P buffer, injected to the chip at 10 μL/min for 120 s contact time. Affinity Constant (KD) was determined with T200 Biacore Software, providing accurate kinetic analysis.

#### IAV-challenged mice experiment

For mice survival and weight evaluation, 4 LD_50_ (median lethal dose) IAV virus was intranasally administered. For lung collection, 2 LD_50_ IAV (H1N1, H3N2, and H5N1) viruses were intranasally administered. After infection, gradient concentration of DHA or vehicle (90% corn oil and 10% DMSO) was intraperitoneally administered for 6 consecutive days. Body weight and survival time were monitored for 16 consecutive days. For lung collection, mice were sacrificed on day 6, and the lung tissues were collected for following experiments.

#### Histopathological assessment

Lung tissues from mice experiments were fixed in 4% polyformaldehyde, embedded in wax, and sectioned every 5 µm serially. Subsequently, the sections underwent deparaffinization and were subjected to standard H&E for evaluating inflammatory infiltration and cell necrosis. All histology analyzes were performed in a double-blinded manner.

#### RNA isolation and qRT-PCR

RNA was isolated with the RNA Easy Fast Tissue/cell Kit (TIANGEN) following the recommended instructions. Then cDNA was obtained by reverse transcription with the Fasting gDNA Dispelling RT SuperMix Fastking kit (TIANGEN) according to manufacturer’s protocol on a T100tm thermal cycler (Bio‐Rad) and then processed for qRT-PCR using a Talent qPCR PreMix (SYBR Green) kit (TIANGEN). Target gene expression was normalized to actin expression and quantified with the 2^-∆∆Ct^ method. Primer sequences are listed in Table S3.

#### Western blotting

Lung tissues from mice experiments or cultured cells were homogenized in RIPA (Beyotime) buffer containing proteinase inhibitors. Then centrifuged at 12,000 rpm for 30 min at 4 °C, and supernatant was collected and measured using BCA Protein Assay Kit (Beyotime). A total of 20 μg extracted protein was separated by 10% SDS-PAGE and transblotted to a PVDF membrane (Millipore). The membrane was blocked and incubated with primary antibodies at 4 °C overnight. Then the membrane was incubated with secondary antibodies for 2 h, and visualized using the Fusion FX Western Blot Imager (Vilber Lourmat).

#### Widely targeted metabolites profiling of patients’ plasma

The sample stored at −80 °C refrigerator was thawed on ice and vortexed for 10 s. 50 μL of sample and 300 μL of extraction solution (ACN: Methanol = 1:4, V/V) containing internal standards were added into a 2 mL microcentrifuge tube. The sample was vortexed for 3 min and then centrifuged at 12,000 rpm for 10 min (4 °C). 200 μL of the supernatant was collected and placed in −20 °C for 30 min, and then centrifuged at 12,000 rpm for 3 min (4 °C). A 180 μL aliquots of supernatant were transferred for LC-MS analysis.

The sample extracts were analyzed using an LC-ESI-MS/MS system (UPLC, ExionLC AD, https://sciex.com.cn/; MS, QTRAP® System, https://sciex.com/). The analytical conditions were as follows, UPLC: column, Waters ACQUITY UPLC HSS T3 C18 (1.8 µm, 2.1 mm*100 mm); column temperature, 40 °C; flow rate, 0.4 mL/min; injection volume, 2 μL; solvent system, water (0.1% formic acid): acetonitrile (0.1% formic acid); solvent B gradient program, 5% to 20% in 2 min, increased to 60% in the following 3 min, increased to 99% in 1 min and held for 1.5 min, then come back to 5% within 0.1 min, held for 2.4 min.

LIT and triple quadrupole (QQQ) scans were acquired on a triple quadrupole-linear ion trap mass spectrometer (QTRAP), QTRAP® LC-MS/MS System, equipped with an ESI Turbo Ion-Spray interface, operating in positive and negative ion mode and controlled by Analyst 1.6.3 software (Sciex). The ESI source operation parameters were as follows: source temperature 500 °C; ion spray voltage (IS) 5500 V (positive), −4500 V (negative); ion source gas I (GSI), gas II (GSII), curtain gas (CUR) were set at 55, 60, and 25.0 psi, respectively; the collision gas (CAD) was high. Instrument tuning and mass calibration were performed with 10 and 100 μmol/L polypropylene glycol solutions in QQQ and LIT modes, respectively. A specific set of MRM transitions was monitored for each period according to the metabolites eluted within this period.

For two-group analysis, differential metabolites were determined by *P* (*P* < 0.05, Student’s *t*-test). The data was log transformed and mean centering before OPLS-DA. In order to avoid overfitting, a permutation test (200 permutations) was performed. Identified metabolites were annotated using KEGG Compound database, annotated metabolites were then mapped to KEGG Pathway database. Pathways with significantly regulated metabolites mapped to were then fed into MSEA (metabolite sets enrichment analysis), and their significance was determined by hypergeometric test’s P-values.

#### Proteome profiling of patients’ plasma

The samples were removed and thawed on ice, and a final concentration of 1 mM PMSF was added to the samples and mixed well by vortex shaking. Then, the samples were enriched for low-abundance proteins in blood by nanomagnetic beads using EasyPeptTM kit (Shanghai eCalculation Biotechnology). Next, reductive alkylation was performed on the magnetic beads, and finally, the proteins were digested into peptides by trypsin. Finally, the peptides were desalted on a C18 column (Millipore), and the peptide concentration was determined by a BCA kit.

Samples were separated using the Vanquish Neo UHPLC liquid chromatography system. The mobile phase A consisted of 0.1% formic acid aqueous solution, while mobile phase B was acetonitrile containing 0.1% formic acid. The injection mode employed a trap-and-elute dual-column method, with a PepMap Neo Trap Cartridge (300 μm * 5 mm, 5 μm) as the trapping column and an Easy-Spray™ PepMap™ Neo UHPLC column (150 μm × 15 cm, 2 μm) as the analytical column. The column temperature was controlled at 55 °C, with an injection volume of 200 ng, a flow rate of 2.5 μl/min, an effective gradient of 6.9 min, and a total runtime of 8 min.

For DIA (Data-Independent Acquisition) analysis, the Vanquish Neo system (Thermo Fisher Scientific) was used for chromatographic separation. Samples separated by nano-flow high-performance liquid chromatography were subjected to DIA mass spectrometry analysis using the Orbitrap Astral high-resolution mass spectrometer (Thermo Scientific). The detection mode was positive ion mode, with a precursor ion scan range of 380–980 m/z, a primary mass resolution of 240,000 at 200 m/z, a Normalized AGC Target of 500%, and a Maximum IT of 5 ms. MS2 was performed using DIA data acquisition mode, with 299 scan windows, an Isolation Window of 2 Th, HCD Collision Energy of 25%, a Normalized AGC Target of 500%, and a Maximum IT of 3 ms.

The MS raw data were analyzed using DIA-NN via the library-free method. The UniProtKB proteome database (UP000005640_human_82493_20240528.fasta) was employed to generate a spectral library through the application of deep learning algorithms based on neural networks. This database encompasses a total of 82,493 sequences. The Match Between Runs (MBR) option was utilized to construct a spectral library from the DIA data, which was subsequently employed for reanalysis. The false discovery rate (FDR) of the search results was stringently controlled to be less than 1% at both the protein and precursor ion levels. Only the identifications that met this stringent criterion were retained for further quantification analysis. To identify proteins with significant changes in abundance, Student’s *T*-Test was applied. The significance threshold was set at an *P* < 0.05, coupled with a fold change > 1.5 or < 0.6667.

#### Quantification and statistical analysis

Data with error bars are expressed as mean ± SD. Statistical analysis of significance between two groups was performed with a student two-sided *T*-test. For comparing multiple groups, one-way ANOVA followed by Tukey’s post hoc comparison was performed. The diagnostic accuracy of plasma metabolites was examined with ROC. Spearman rank correlation was applied to identify purine nucleotide metabolites significantly correlated with plasma cytokines of H1N1-positive patients. The associations of metabolites and plasma cytokines were determined by linear regression analysis. Differences were determined statistically significant if *P* < 0.05.

## Supplementary information

SUPPLEMENTAL MATERIAL

Raw WB

Table S1. VI-PPI

Table S2. SAFE cluster result

## Data Availability

RNA sequencing data are publicly available from the China National Gene Bank (accession number CNP0005904). Other data has been deposited at Mendeley (https://data.mendeley.com/datasets/pbp2sw6gg3/).

## Materials availability

All unique reagents generated in this study will be provided available by the lead contact.
