# Supplementary material for: Purine nucleoside phosphorylase dominates Influenza A virus replication and host hyperinflammation through purine salvage
Source: Signal Transduct Target Ther. 2025 Jun 15;10:191. doi: 10.1038/s41392-025-02272-1 (PMC12167387; doi:10.1038/s41392-025-02272-1)
Supplement: Supplementary file 1 — SUPPLEMENTAL MATERIAL [file 41392_2025_2272_MOESM1_ESM.docx]

Supplementary Materials for

**Purine nucleotide phosphorylase dominates IAV replication and host hyperinflammation through purine salvage**

Yang Yue, Qingyu Li, Changguo Chen, Juntao Yang, Weian Song, Changdong Zhou, Yuke Cui, Zhenqiao Wei, Qi He, Chenhui Wang, Hongjun Lin, Jiangbo Li, Jian Li, Ji Xi, Xiang Song, Wen Yang, Ze Zhang, Wenjie Shu, Liang Guo, Shengqi Wang

Correspondence to: sqwang@bmi.ac.cn, [gllsunmo369@sina.com](mailto:gllsunmo369@sina.com), shuwj@bmi.ac.cn.

**This PDF file includes:**

Figures. S1 to S8

Tables S3 to S5

**Other Supplementary Materials for this manuscript include the following:**

Table S1 to Table S2


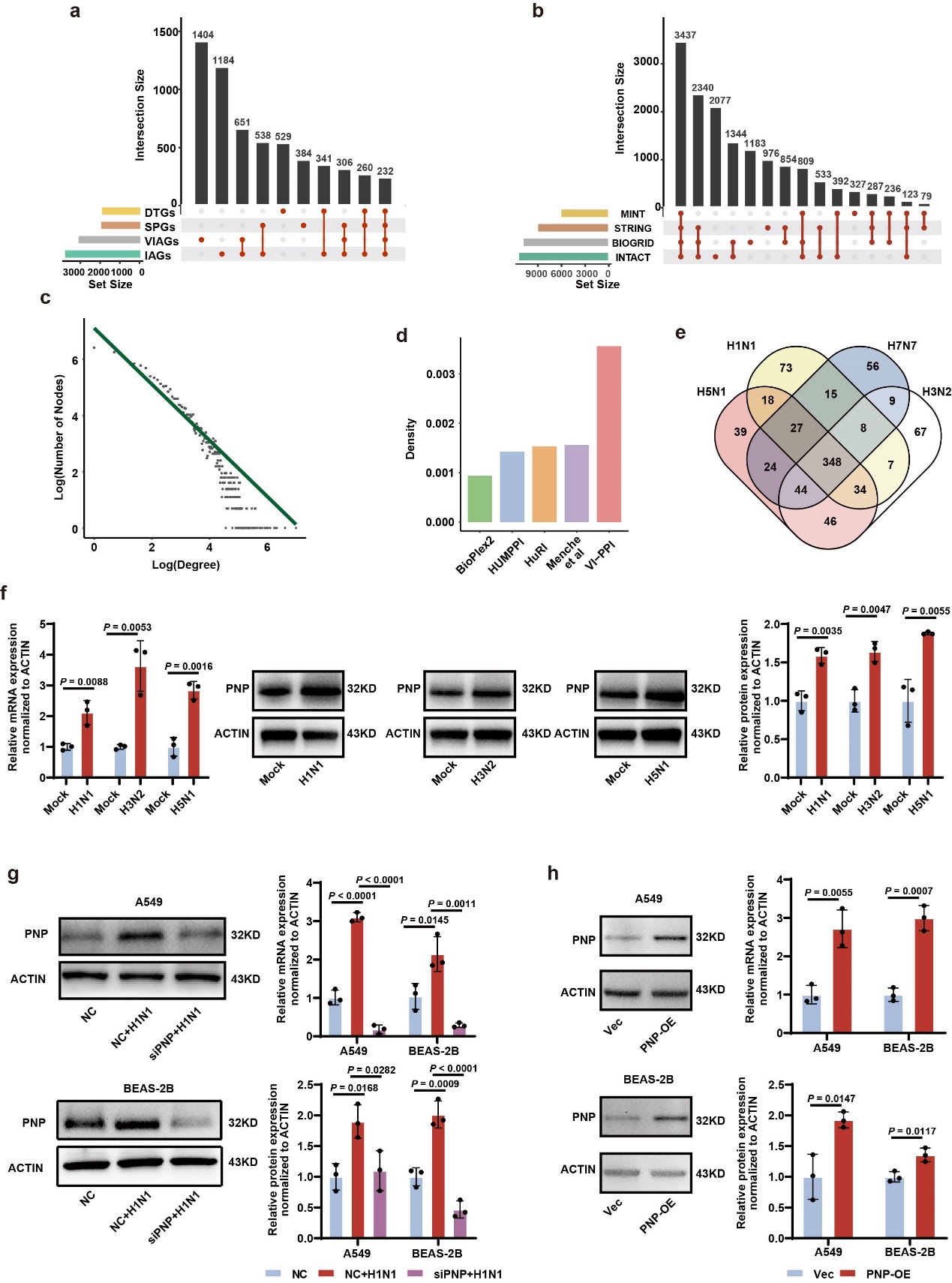


**Supplementary Fig. 1**

**(a)** The UpSetR plots of the different types of collected protein-coding genes including inflammation-associated genes (IAGs), viral-infection-associated genes (VIAGs), signaling-pathway-associated gene (SPAGs), drug-target genes (DTGs).

**(b)** The UpSetR plots of the PPIs extracted from MINT, STRING, BIOGRID, and INTACT databases.

**(c)** Power-law degree distribution showed the scale-free property of VI-PPI network.

**(d)** Comparison of VI-PPI to other PPI networks by the edge density. The edge density was defined as the proportion of the count of edges in the total possible edges of each PPI network.

**(e)** Venn plot for overlapping hub genes among the 4 different strains of influenza virus (H1N1, H7N7, H5N1, H3N2).

**(f)** The relative mRNA and protein level of PNP in the Lungs of Mice Infected with H1N1, H3N2, and H5N1.

**(g)** The relative mRNA and protein level of PNP in A549 and BEAS-2B cells treated by NC siRNA or PNP siRNA with or without H1N1 infection.

**(h)** The relative mRNA and protein level of PNP in A549 and BEAS-2B cells treated by control vector and PNP-overexpression plasmid with or without H1N1 infection.

All data are presented as $\text{mean}\text{ }\text{±}\text{ }\text{SD}$; N = 3 biologically independent experiments; Statistical analysis was performed by Two-way ANOVA (**f-h**).


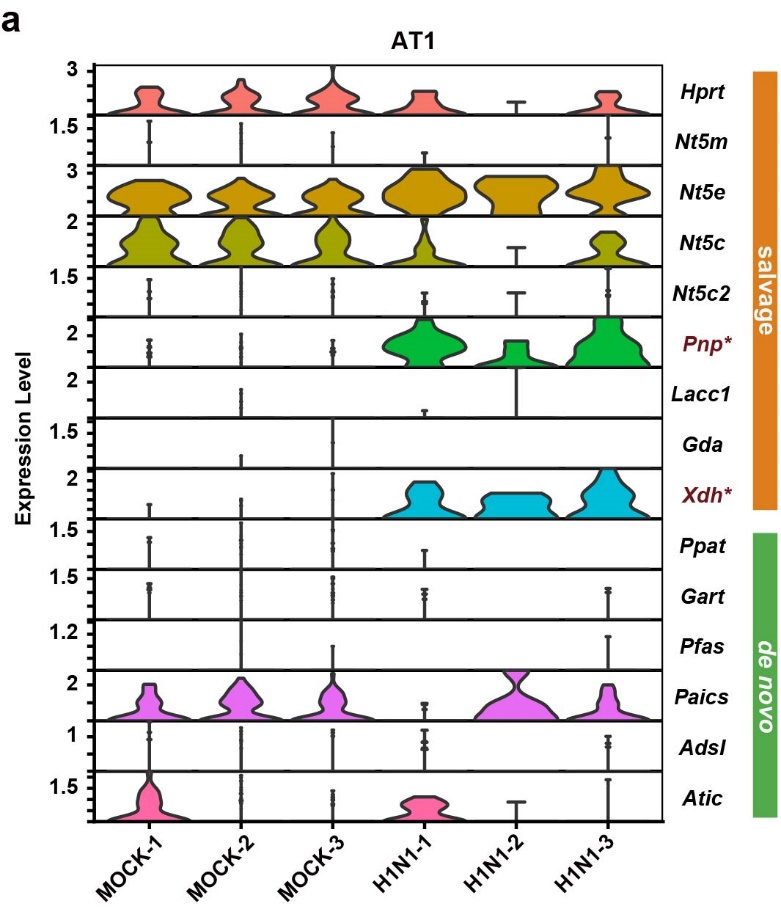


**Supplementary Fig. 2**

**(a)** The stacked violin plot of enzymes involved in purine de novo and salvage pathway by scRNA-seq data. The scRNA-seq data (CRA013573, CNCB) of the control and H1N1-challenged mice were used to analyze the expression of the enzymes functioning in purine metabolism.


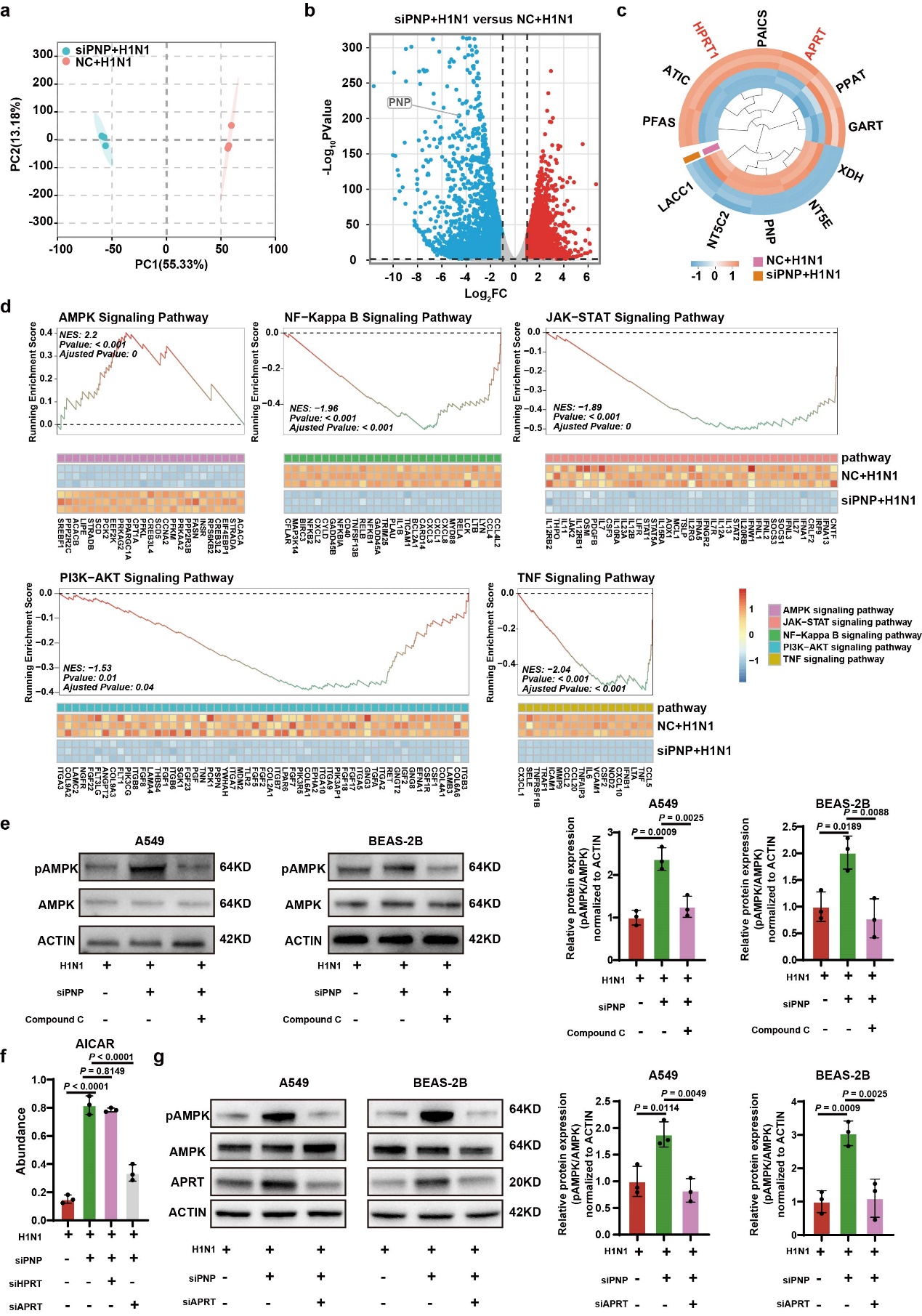


**Supplementary Fig. 3**

**(a)** PCA assay for each sample of the transcriptomic sequencing for A549 cell transfected by NC siRNA or PNP siRNA upon H1N1 challenge.

**(b)** Volcano plot of DEGs in H1N1-infected PNP-knockout A549 (siPNP+H1N1) versus infected cells (NC+H1N1). The up‐regulated genes (red) and down‐regulated genes (blue) with a |log_2_(fold change)| > 1, and with *P* < 0 .05 were indicated in the plot.

**(c)** Heatmap of critical enzymes of purine synthesis. The expression of enzymes involved in purine salvage and *de novo* pathways were represented by heatmap based on DEGs of siPNP+H1N1 vs NC+H1N1.

**(d)** GSEA analysis of significantly enriched signaling pathways based on DEGs derived from NC+H1N1 vs siPNP+H1N1. The core genes of each enriched pathway, colored by fold change, were displayed.

**(e)** Westen blot analyses of AMPK activation. PNP-knockdown A549 and BEAS-2B cells were treated with or without Compound C (10 µM) upon H1N1 (MOI=5). Total AMPK, p-AMPK, and Actin were analyzed by Western blot.

**(f)** Abundance of AICAR in A549 cells treated by NC siRNA+H1N1, PNP siRNA+H1N1, PNP siRNA+ HPRT siRNA+H1N1 and PNP siRNA+ APRT siRNA+H1N1based on the LC-MS.

**(g)** Westen blot analyses of AMPK activation. PNP-knockdown A549 and BEAS-2B cells were transfected with APRT upon H1N1 (MOI = 5). Total AMPK, p-AMPK, and Actin were analyzed by Western blot.

All data are presented as $\text{mean}\text{ }\text{±}\text{ }\text{SD}$; N = 3 biologically independent experiments; Statistical analysis was performed by one-way ANOVA (**e-g**).


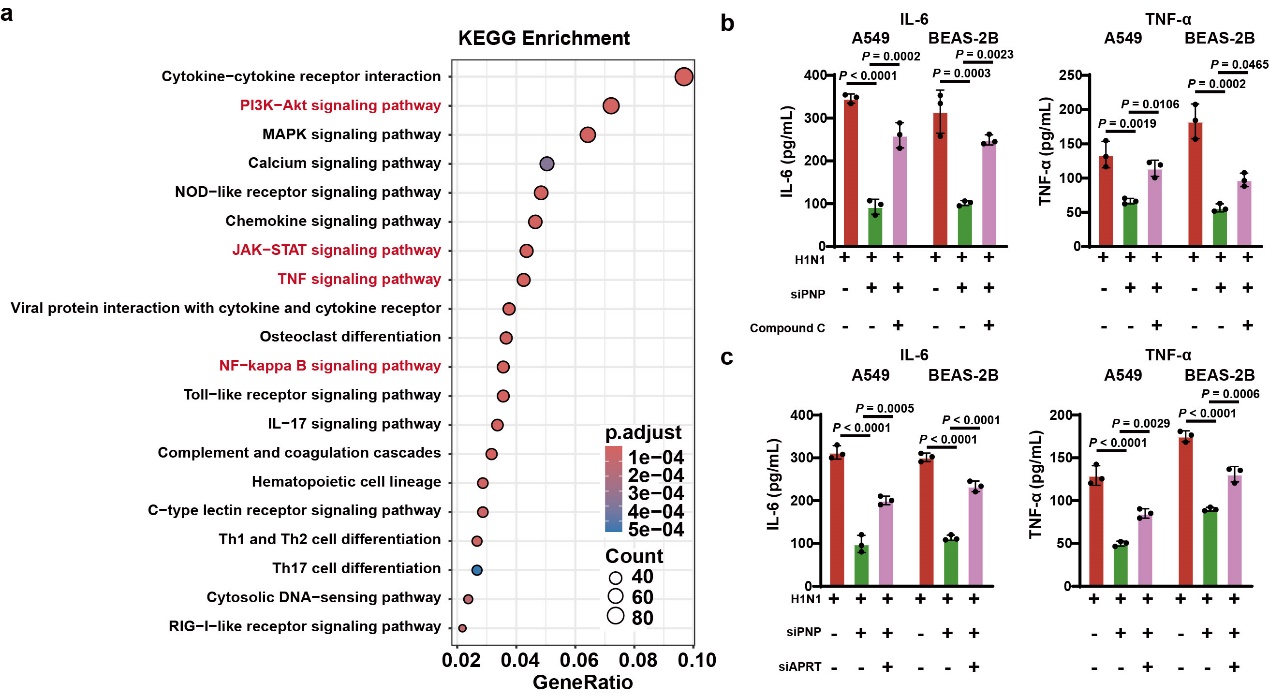


**Supplementary Fig. 4**

**(a)** Dot plot for KEGG pathway enrichment analysis based on DEGs of siPNP/H1N1 vs NCsiRNA/H1N1. Inflammatory pathways were colored in red.  Dot size represents the number of DEGs in each KEGG pathway; *P* adjust (adjusted *P*): Red < purple < blue; pathways with adjusted *P* < 0.05 were considered significant.

**(b)** TNF-ɑ and IL-6 levels in A549 and BEAS-2B supernatant were measured by ELISA. PNP-knockdown A549 and BEAS-2B cells were treated with or without Compound C (10 µM) for 24 hours upon H1N1 (MOI = 5).

**(c)** TNF-ɑ and IL-6 levels in A549 and BEAS-2B supernatant were measured by ELISA. PNP-knockdown A549 and BEAS-2B cells were transfected with APRT upon H1N1 (MOI = 5).

All data are presented as $\text{mean}\text{ }\text{±}\text{ }\text{SD}$; N = 3 biologically independent experiments; Statistical analysis was performed by Two-way ANOVA (**b-c**).


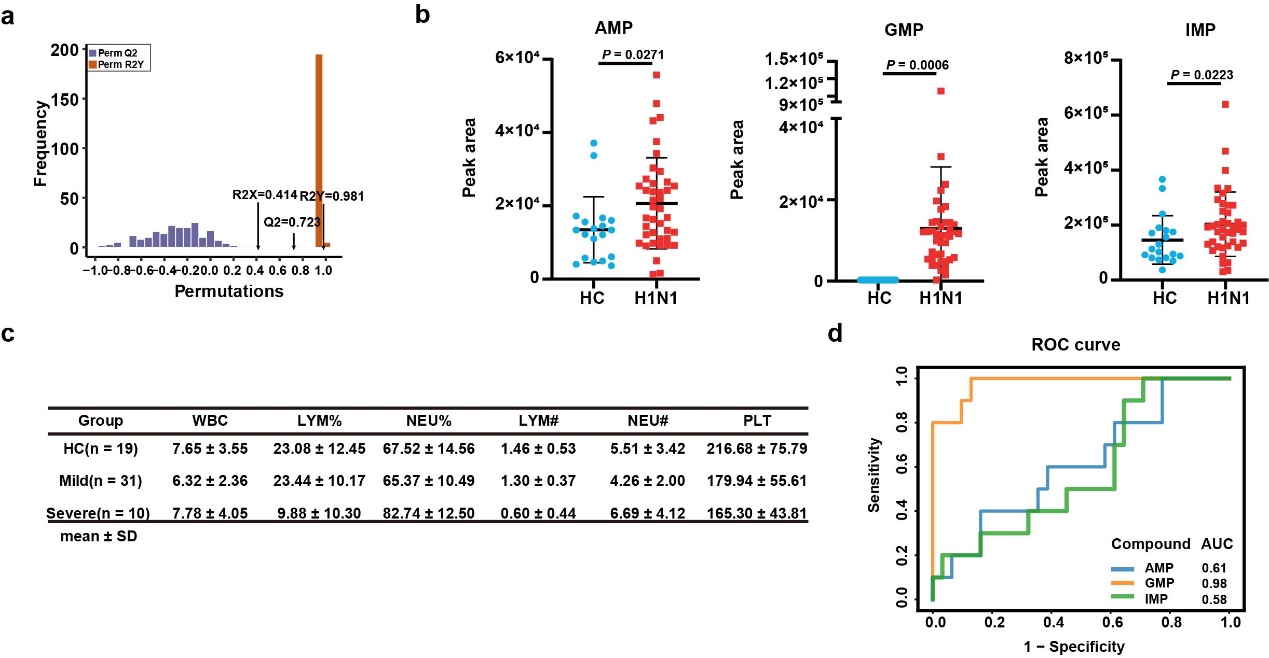


**Supplementary Fig. 5**

**(a)** OPLS-DA validation of the widely-targeted metabolomic data of H1N1-infected and healthy individuals with permutation (R2X = 0.414, R2Y = 0.981, Q2 = 0.723). The discriminant model displayed significant separation between H1N1-positive and negative groups.

**(b)** Comparison of peripheral purine metabolites between healthy controls and H1N1-infected individuals by widely-targeted metabolomic data.

**(c)** The hematological parameters of the participants enrolled in the study. WBC: White Blood Cell Count; LYM%: Lymphocyte Percentage; NEU%: Neutrophil Percentage; LYM#: Lymphocyte Absolute Count; NEU#: Neutrophil Absolute Count; PLT: Platelet Count;

**(d)** AUC-ROC curve analysis of the correlation between purine nucleotide and disease progression. The discriminative capability of purine nucleotide GMP, IMP, and AMP in distinguishing between individuals with mild and severe conditions was quantified by area under ROC curve (AUC), with GMP showing the highest AUC.

All data are presented as $\text{mean}\text{ }\text{±}\text{ }\text{SD}$; Statistical analysis was performed by two-tailed Student’s t test (**b**).


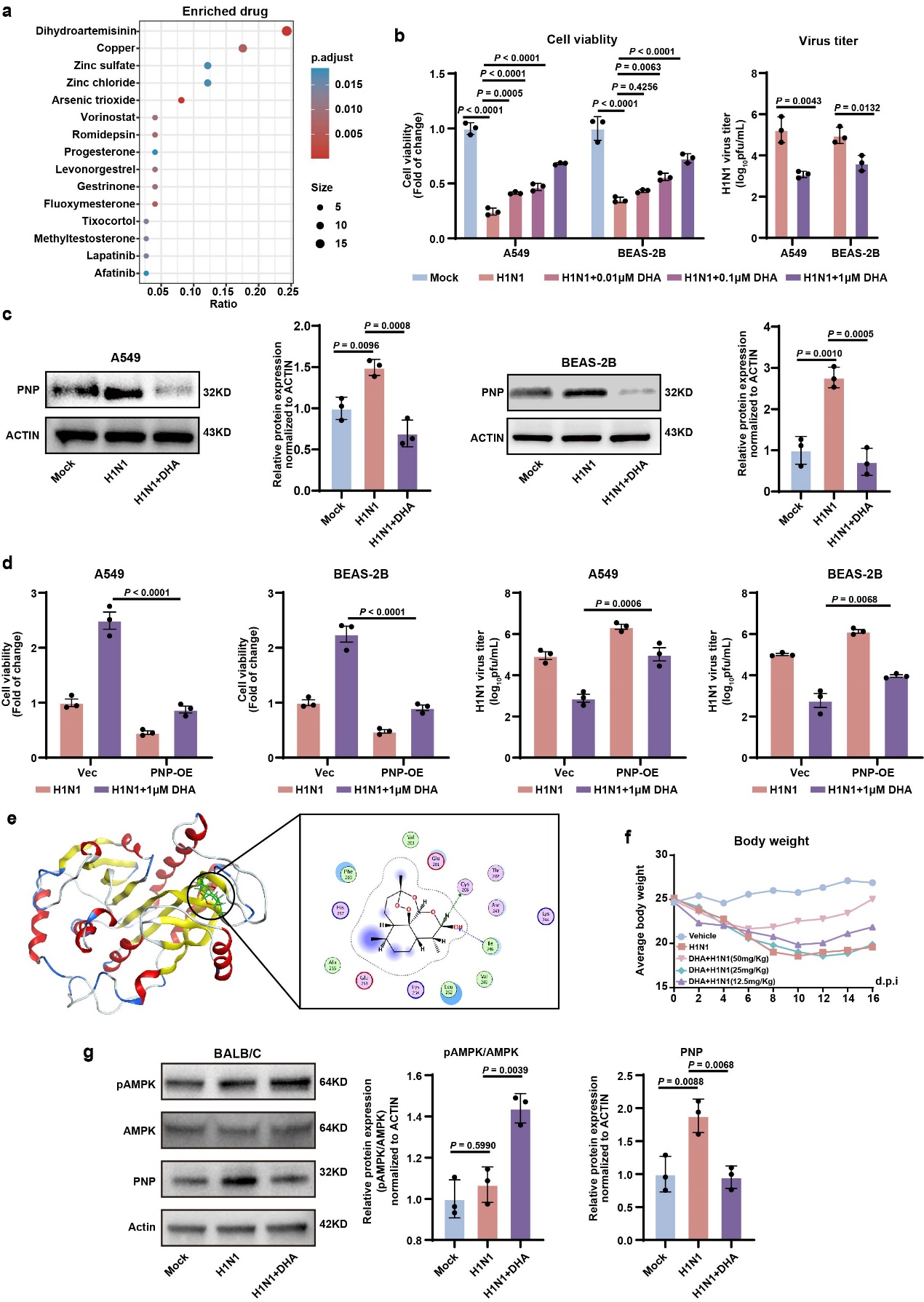


**Supplementary Fig. 6**

**(a)** The dot plot for the enrichment analysis of potential PNP-targeting compounds predicted by VI-PPI network. The top 15 compounds are shown.

**(b)** Cell viability and virus titer of H1N1-infected A549 and BEAS-2B cells under the treatment of dihydroartemisinin at different doses (0.01, 0.1, 1 μM) for 24 hours.

**(c)** The inhibitory effect of dihydroartemisinin on PNP in AECs upon H1N1 infection. A549 and BEAS-2B cells were infected by H1N1 (MOI = 5) for 3 hours, and then treated with 1μM dihydroartemisinin for another 24 hours. The relative and protein expression of PNP in the control, infected, and dihydroartemisinin -treated groups were measured by Western blot analysis.

**(d)** PNP-overexpressed A549 and BEAS-2B cells were treated with or without DHA (1μM) upon H1N1, followed by cell viability analysis (MOI = 5).

**(e)** Docking of DHA in the active site of PNP (left), and 2-D depiction of the docking conformation between DHA and the active binding site of PNP (right). The hydrogen bonds were marked by dotted lines.

**(f)**The body weight change was monitored for 16 consecutive days after infection.

**(g)** Western blot analysis of AMPK activation and PNP expression in mice models treated with DHA. Control and H1N1-challenged mice were treated with DHA for a consecutive 6 days, followed by lung dissection. The lungs were lysed for Westen blot analyses to examine the expression of AMPK, p-AMPK, PNP, and ACTIN.

All data are presented as $\text{mean}\text{ }\text{±}\text{ }\text{SD}$; unless otherwise indicated, N = 3 biologically independent experiments; Statistical analysis was performed by one-way ANOVA (**c**, **g**); Two-way ANOVA in **b** and **d**.


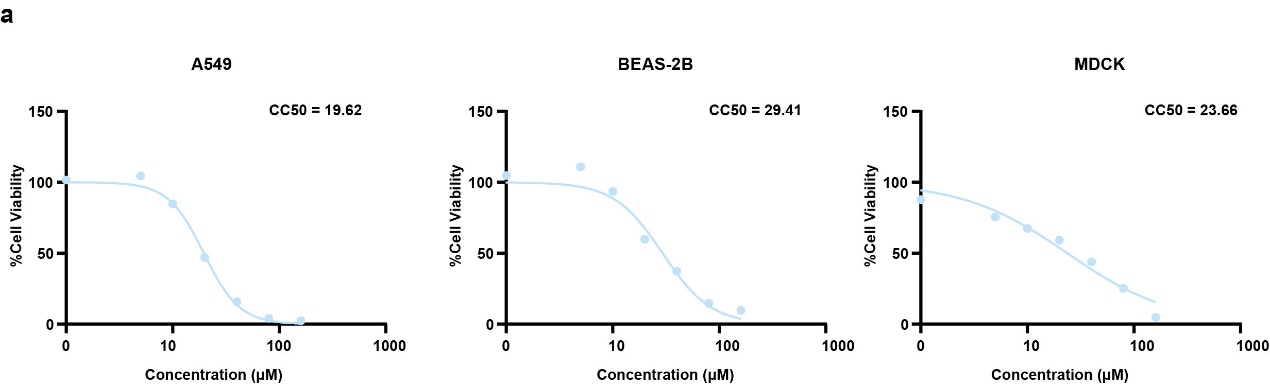


**Supplementary Fig. 7**

**(a)** CC50 of DHA in A549, BEAS-2B, and MDCK cells.


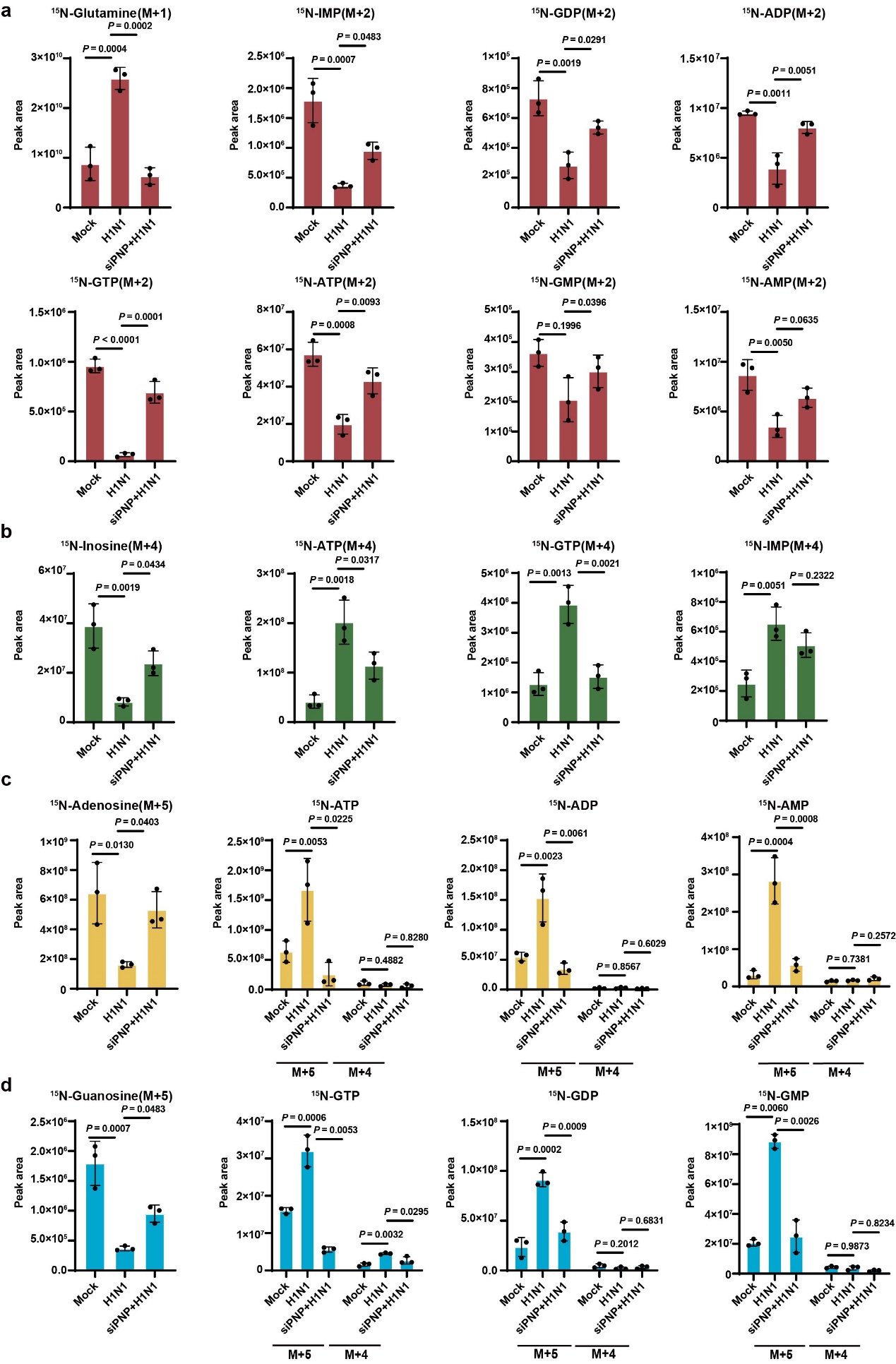


**Supplementary Fig. 8.**

The control and H1N1-infected BEAS-2B cells were cultured in media containing ^15^N-labeled starting material for 4 hours before mass spectrometry analysis of isotype-labeled glutamine **(a)**, inosine **(b)**, adenosine **(c)**, and guanosine **(d)**, as well as related purine nucleosides. The experiment was conducted in triplicate. M+(n): the gross (n) of incorporated ^15^N.

All data are presented as $\text{mean}\text{ }\text{±}\text{ }\text{SD}$; unless otherwise indicated, N = 3 biologically independent experiments; Statistical analysis was performed by one-way ANOVA (**a**, **b**), **c** (^15^N-Adenosine) and **d** (^15^N-Guanosine); Two-way ANOVA in **c** (^15^N-ATP, ^15^N-ADP and ^15^N-AMP) and **d** (^15^N-GTP, ^15^N-GDP and ^15^N-GMP).

| **Table S3 List of qRT-PCR primer sequences** | | |
| --- | --- | --- |
| Name | Primer sequence | |
|  | F (5’-3’) | R (5’-3’) |
| PNP (homo sapiens) | TTTCCCCGAAGTACAGTGCC | CCCTCACTGGGAATGTCACC |
| ACTIN (homo sapiens) | AGGCACCAGGGCGTGA | CGATGGGGTACTTCAGGGTG |
| NP(H1N1) | CTCATCCTTTATGACAAAGAAG | AGATCATCATGTGAGTCAGAC |
| PNP (mouse) | CCCAACGATGAAAGGTTTGGAG | GGCCTGCCAACATCACGTA |
| ACTIN (mouse) | CACCCTGTGCTGCTCACC | GAAGGTCTCAAACATGATCTGGGT |
| PB1-F2 ChIP-PCR | GAGGTGATTCACAGTTGGA | TTAGTAGAGACGGGGTTTT |

| **Table S4. Study design and patients** | | |
| --- | --- | --- |
|  | **H1N1 Positive** | **Healthy Control** |
| **Sex** |  | |
| Female | 21 | 9 |
| Male | 20 | 10 |
| **Age** | 73.3 | 71.5 |
| Mean (SD) | (25.5) | (22.9) |
| Sample number | (41) | (19) |

**Table S5 Clinical Symptoms of Participants**

| **Participants** | **Clinical symptoms** | **Disease severity** |
| --- | --- | --- |
| H1N1_1 | Mild cough, mild fatigue, runny nose | Mild |
| H1N1_10 | Mild cough, low-grade fever (37.5°C-38.5°C), mild fatigue | Mild |
| H1N1_11 | Mild cough, low-grade fever (37.5°C-38.5°C), mild fatigue | Mild |
| H1N1_12 | High fever (≥39°C), severe cough, chest pain | Severe |
| H1N1_13 | Low-grade fever (37.5°C-38.5°C), mild fatigue | Mild |
| H1N1_14 | Severe respiratory distress, high fever (≥39°C), severe cough, chest pain, ARDS | Severe |
| H1N1_15 | Mild cough, low-grade fever (37.5°C-38.5°C), mild fatigue | Mild |
| H1N1_16 | Mild cough, low-grade fever (37.5°C-38.5°C), mild fatigue | Mild |
| H1N1_17 | Mild cough, low-grade fever (37.5°C-38.5°C), mild fatigue | Mild |
| H1N1_18 | Low-grade fever (37.5°C-38.5°C), mild fatigue | Mild |
| H1N1_19 | High fever (≥39°C), severe cough | Severe |
| H1N1_2 | Mild cough, low-grade fever (37.5°C-38.5°C) | Mild |
| H1N1_20 | Mild cough, low-grade fever (37.5°C-38.5°C), mild fatigue | Mild |
| H1N1_21 | Mild cough, low-grade fever (37.5°C-38.5°C), mild fatigue | Mild |
| H1N1_22 | Mild cough, low-grade fever (37.5°C-38.5°C), mild fatigue, sore | Mild |
| H1N1_23 | Mild cough, low-grade fever (37.5°C-38.5°C), mild fatigue | Mild |
| H1N1_24 | Mild cough, low-grade fever (37.5°C-38.5°C), mild fatigue, sore throat, runny nose | Mild |
| H1N1_25 | Mild cough, low-grade fever (37.5°C-38.5°C), mild fatigue, sore throat, runny nose | Mild |
| H1N1_26 | Mild fatigue, sore throat, runny nose | Mild |
| H1N1_27 | Mild fatigue, sore throat, runny nose | Mild |
| H1N1_28 | Low-grade fever (37.5°C-38.5°C), mild fatigue | Mild |
| H1N1_29 | Severe respiratory distress, high fever (≥39°C) | Severe |
| H1N1_3 | ARDS | Severe |
| H1N1_30 | Mild cough, low-grade fever (37.5°C-38.5°C), mild fatigue, sore throat, runny nose | Mild |
| H1N1_31 | Mild cough, low-grade fever (37.5°C-38.5°C), mild fatigue | Mild |
| H1N1_32 | Low-grade fever (37.5°C-38.5°C), mild fatigue | Mild |
| H1N1_33 | Mild cough, mild fatigue, runny nose | Mild |
| H1N1_34 | Mild cough, low-grade fever (37.5°C-38.5°C), mild fatigue | Mild |
| H1N1_35 | Mild cough, low-grade fever (37.5°C-38.5°C), mild fatigue | Mild |
| H1N1_36 | High fever (≥39°C), severe cough | Severe |
| H1N1_37 | Mild cough, mild fatigue, runny nose | Mild |
| H1N1_38 | Mild cough, low-grade fever (37.5°C-38.5°C) | Mild |
| H1N1_39 | High fever (≥39°C), severe cough | Severe |
| H1N1_4 | Severe respiratory distress | Severe |
| H1N1_40 | Mild cough, low-grade fever (37.5°C-38.5°C), mild fatigue | Mild |
| H1N1_41 | Mild cough, mild fatigue, runny nose | Mild |
| H1N1_5 | Chest pain, ARDS | Severe |
| H1N1_6 | Mild cough, low-grade fever (37.5°C-38.5°C) | Mild |
| H1N1_7 | Severe respiratory distress, high fever (≥39°C), severe cough, chest pain, ARDS | Severe |
| H1N1_8 | Mild cough, low-grade fever (37.5°C-38.5°C), mild fatigue, sore throat, runny nose | Mild |
| H1N1_9 | Mild cough, mild fatigue, runny nose | Mild |
| HC1 | \ | \ |
| HC10 | \ | \ |
| HC11 | \ | \ |
| HC12 | \ | \ |
| HC13 | \ | \ |
| HC14 | \ | \ |
| HC15 | \ | \ |
| HC16 | \ | \ |
| HC17 | \ | \ |
| HC18 | \ | \ |
| HC19 | \ | \ |
| HC2 | \ | \ |
| HC3 | \ | \ |
| HC4 | \ | \ |
| HC5 | \ | \ |
| HC6 | \ | \ |
| HC7 | \ | \ |
| HC8 | \ | \ |
| HC9 | \ | \ |
