# Supplementary material for: Purine nucleoside phosphorylase dominates Influenza A virus replication and host hyperinflammation through purine salvage
Source: Signal Transduct Target Ther. 2025 Jun 15;10:191. doi: 10.1038/s41392-025-02272-1 (PMC12167387; doi:10.1038/s41392-025-02272-1)
Supplement: Supplementary file 2 — Raw WB [file 41392_2025_2272_MOESM2_ESM.docx]

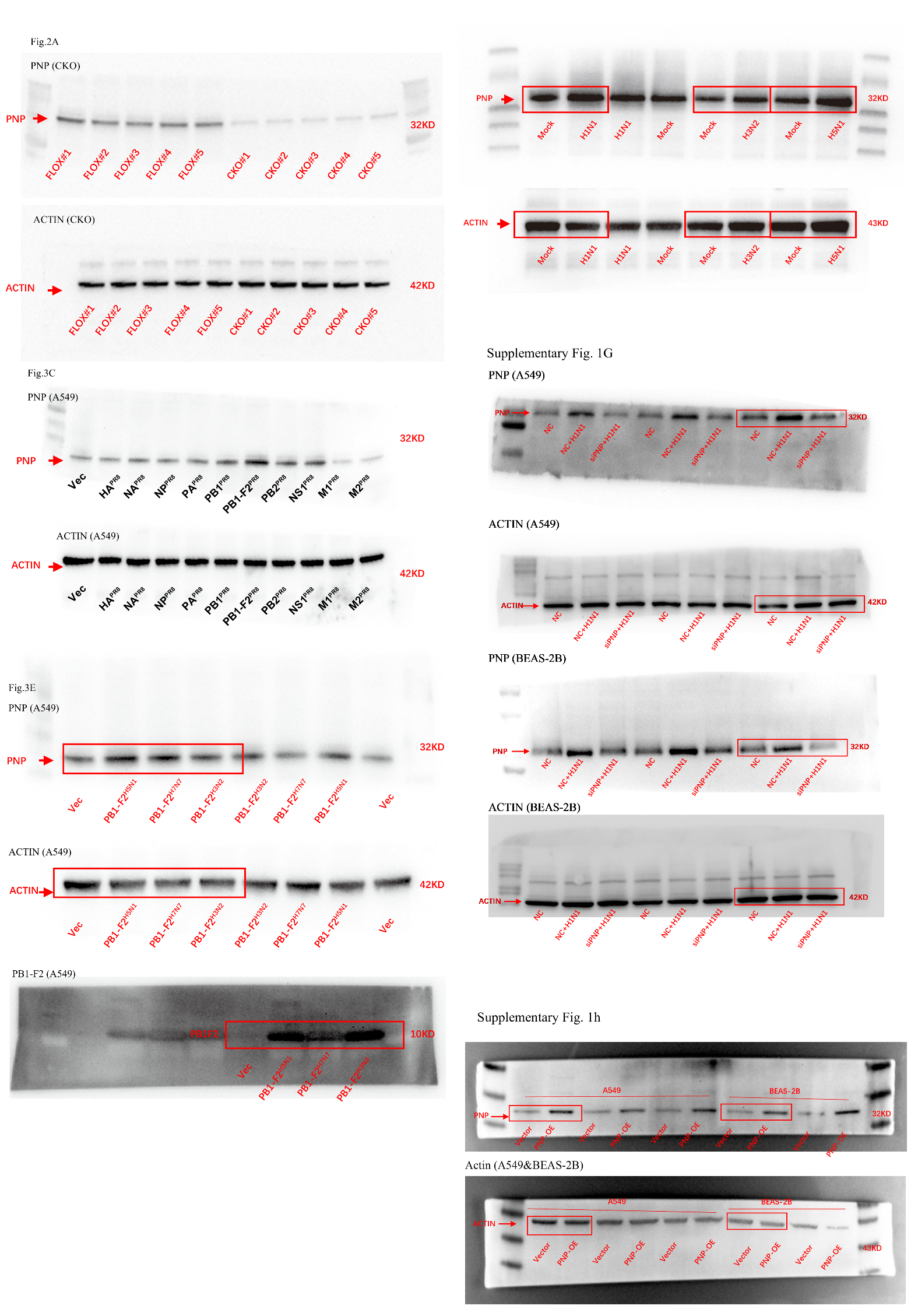


**Supplementary Fig. 1g**

**Fig. 3e**

**Supplementary Fig. 1h**

**Supplementary Fig. 1f**

**Fig. 2a**


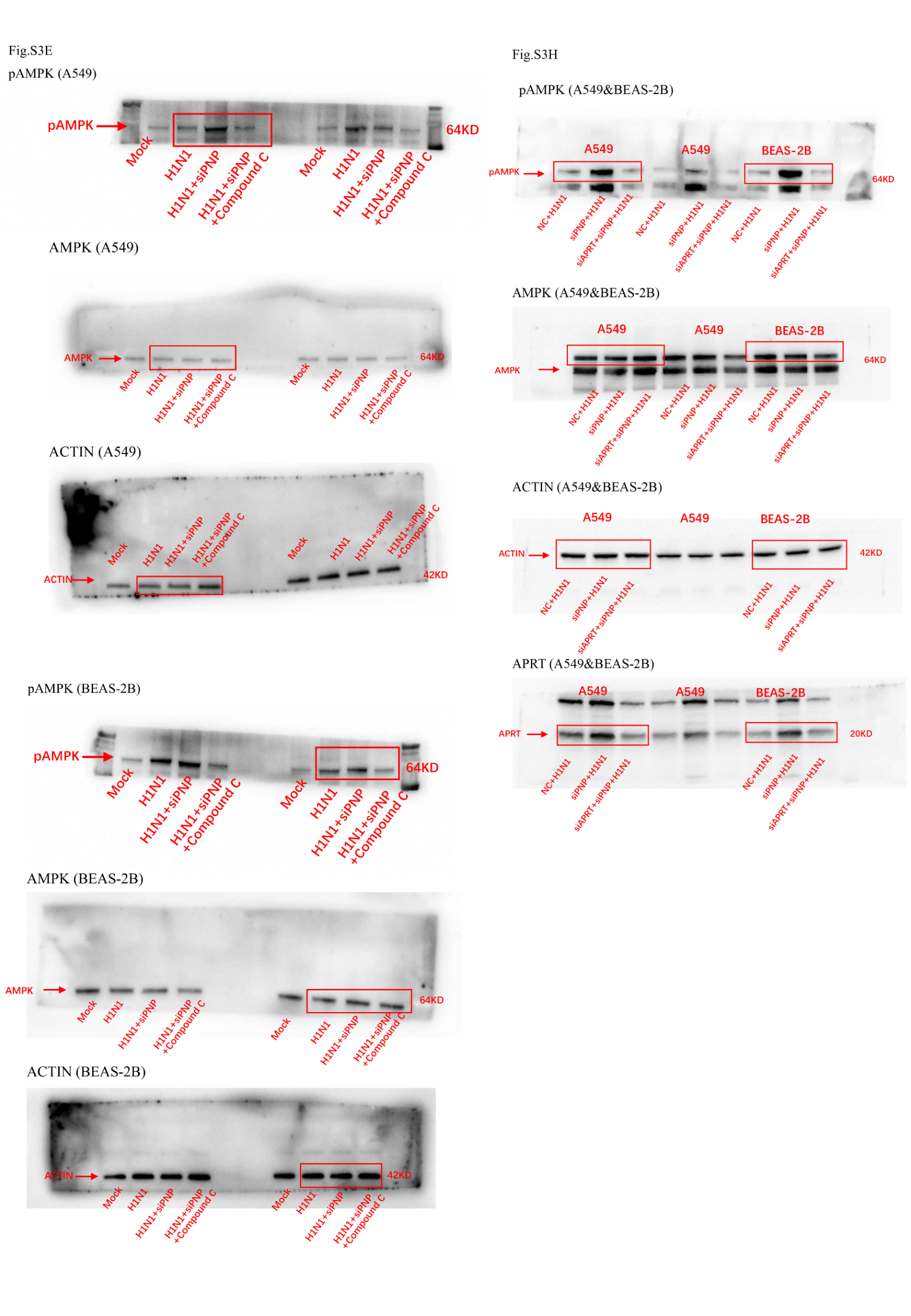


**Supplementary Fig. 3h**

**Supplementary Fig. 3e**


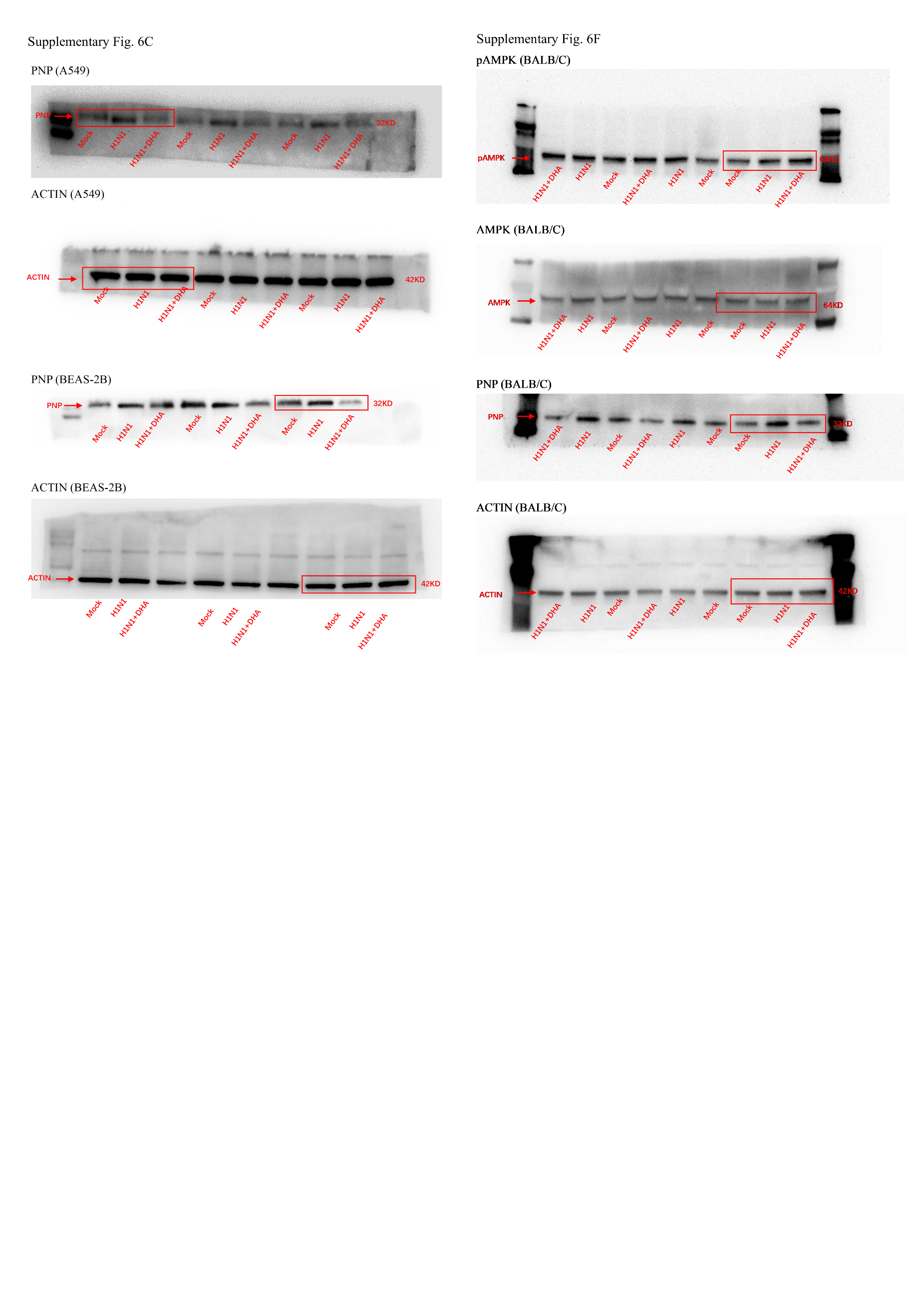


**Supplementary Fig. 6f**

**Supplementary Fig. 6c**
